# Phenotype Classification of Intact Cells by NMR Spectroscopy through Machine Learning Approaches

**DOI:** 10.1021/jacs.6c01100

**Published:** 2026-04-22

**Authors:** Carlo Mengucci, Claudia Dell'Amico, Simona Del Giudice, Letizia Barbieri, Alice Mariottini, Marco Onorati, Luca Massacesi, Enrico Luchinat, Lucia Banci

**Affiliations:** † Department of Agri-Food Science and Technology, 9296 University of Bologna, Piazza Goidanich 60, Cesena 47521, Italy; ‡ Department of Biology, 9310 University of Pisa, via Luca Ghini 13, Pisa 56126, Italy; § Department of Clinical and Experimental Medicine, University of Pisa, via Savi 10, Pisa 56126, Italy; ∥ Magnetic Resonance Center − CERM, 9300 University of Florence, via Luigi Sacconi 6, Sesto Fiorentino 50019, Italy; ⊥ 524266 Interuniversity Consortium for Magnetic Resonance of Metalloproteins − CIRMMP, via Luigi Sacconi 6, Sesto Fiorentino 50019, Italy; # Department of Neurosciences, Psychology, Drug Research and Child Health, 9300 University of Florence, viale Pieraccini 6, Florence 50139, Italy; ∇ Department of Emergency Neurology, Careggi University Hospital, largo Piero Palagi, 1, Florence 50139, Italy; ○ Department of Chemistry “Ugo Schiff”, University of Florence, via della Lastruccia 3, Sesto Fiorentino 50019, Italy

## Abstract

NMR spectroscopy is a powerful, noninvasive tool to analyze complex biological samples. *In vitro*, high-resolution, 1D NMR spectra of biofluids and cell extracts make it possible to classify biological samples based on their metabolic fingerprint. However, such analysis is currently not possible with live cells or tissues, or by spectroscopic imaging *in vivo,* due to the line broadening arising from the intrinsic inhomogeneity of such samples, causing severe signal overlap. Here, we show that machine learning approaches applied to poorly resolved NMR spectra of live, intact cells recorded at high fields allow for the classification of different physiopathologically relevant cell types cultured *in vitro*. We demonstrate the successful classification of neural progenitor cells, neurons, and astrocytes, as well as the classification of mixed cell type samples, and show that a classifier trained on high-field NMR spectra can discriminate cells analyzed at lower fields, approaching those of current MRI instruments. In the future, this approach could be further developed for MRSI data analysis applications, potentially offering a noninvasive diagnostic tool for lesions of the central nervous system and reducing the need for biopsies.

## Graphical abstract

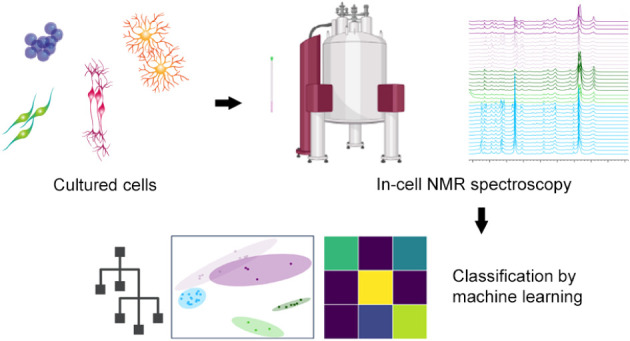

## Introduction

Solution nuclear magnetic resonance (NMR) spectroscopy performed at high magnetic fields (11.7–28.3 T) is a powerful tool for the high-resolution analysis of complex mixtures of biofluids and microenvironments. Indeed, NMR-based metabolomics is a robust approach to obtain quantitative information on tens of metabolites (metabolic profiling) and to discriminate among groups of related samples based on spectral features (metabolic fingerprinting).^1,2^ Furthermore, because of its noninvasive and nondestructive features, NMR is a suitable method to investigate living cells. Indeed, both microorganisms and eukaryotic cells can be investigated by NMR to obtain information on their structural components and chemical composition.^3^ In the past two decades, NMR has also been applied to investigate macromolecules in intact cells. This “in-cell NMR” approach provides unique information about the structure, function, and interactions of biomolecules in their physiological environment.^4,5^ While biological fluids analyzed *in vitro* give rise to highly resolved information-rich spectra, the spectral features arising from metabolites analyzed *in situ* (i.e., in intact cells or tissues) are broadened due to sample inhomogeneity, resulting in overlapping signals even at high magnetic fields. For this reason, *in situ* NMR metabolomic analysis is either performed on diluted cell suspensions^6^ or carried out under high-resolution magic-angle spinning (HR-MAS) conditions to recover spectral resolution.^7^

The broad spectral features found in high-field NMR spectra recorded on static samples of intact cells bear a striking resemblance to those observed *in vivo* by Magnetic Resonance Spectroscopic Imaging (MRSI).^8,9^ MRSI, or Chemical Shift Imaging (CSI), measures NMR spectra as a function of spatial coordinates using a magnetic resonance imaging (MRI) instrument, providing 3D maps of the main soluble chemical components and metabolites in samples such as biofluids, cell lines, vital tissues, or tissue homogenates. Thanks to its noninvasive nature, MRSI is a very promising tool for clinical investigations, such as diagnosis of lesions of the central nervous system (CNS).^10^ However, in clinical practice, the contribution of MRSI to the anatomical and pathological characterization of the CNS has thus far been limited in sensitivity, due to the lower fields of MRI compared to NMR spectrometers, and in resolution, due to the magnetic field inhomogeneity of the sample, which results in highly overlapped spectral features.^11–13^ These drawbacks currently limit the application of MRSI to clinical practice, as this analysis is typically restricted to a few abundant brain metabolites—even at 7 T magnetic field strength—and is therefore still mainly confined to research settings.^14,15^

Several NMR experiments have been reported that can overcome field inhomogeneity and provide highly resolved “solution-like” NMR spectra on inhomogeneous samples such as tissues or living organisms.^16,17^ However, all these approaches gain resolution at the expense of sensitivity and, therefore, are not applicable to the above scenarios. On the other hand, multivariate analysis approaches are able to identify differences within large ensembles of spectra just by exploiting signal intensity variations, without restrictions on the type of NMR experiment and—importantly—regardless of the shapes of the signals. While such approaches are typically employed to analyze high-resolution NMR spectra of biofluids,^18–21^ in principle, they should also be able to extract relevant differences between low-resolution NMR spectra of different types of cells, which arise from specific lipidomic, proteomic, and metabolomic profiles.

In this work, we show that the poorly resolved NMR spectral features of intact cells can be exploited to classify different cell types cultured *in vitro* by using multivariate analysis. We applied machine learning techniques for dimensionality reduction of the spectra and their classification, with the goal of detecting alterations in spectral features arising from the unique fingerprint of each cell type as well as time-related changes occurring in neural stem cells undergoing differentiation. The approach is demonstrated on a large set of live cell samples obtained from cultured HeLa cells, HEK293T cells, Jurkat T lymphocytes, and neural progenitor cells (NPCs). NPCs are multipotent progenitors considered the founder population of the developing human CNS,^22–24^ derived from human-induced pluripotent stem cells (iPSCs). We also included iPS-NPC-derived neurons (hereafter neurons) and iPSC-derived astrocytes (hereafter astrocytes).^22^ We show that multivariate analysis by Partial Least Squares Discriminant Analysis (PLSDA)^25^ and class separation with a Support Vector Machine (SVM)^26^ are excellent classifiers of pure cell types, while feature extraction by Boruta^27^ followed by a Random Forest Classifier (RFC)^28^ can also correctly identify samples containing two cell types mixed together. Furthermore, we demonstrate that a training set recorded at high field can be used to classify samples analyzed at lower field applicable *in vivo* with relatively high accuracy, highlighting the robustness of the approach. Finally, we show that multivariate analysis is sensitive to the changes in phenotype induced by the differentiation of NPCs toward neurons and astrocytes.^29,30^ The successful classification of NPCs, neurons, glial cells, and immune cells, which are representative of key components of the CNS, suggests that this approach might be further developed to allow clinical applications, eventually enabling a “virtual biopsy” of the CNS by MRSI.

## Results and Discussion

### Creation of a Database of NMR Spectra of Intact Cells

To demonstrate that multivariate analysis of NMR spectra recorded on intact cells can be employed to classify different phenotypes, a large number of samples of different human cell types (HEK293T, HeLa, Jurkat T lymphocytes, NPCs, neurons, and astrocytes) were produced and analyzed by in-cell ^1^H NMR at 950 MHz (22.3 T). This resulted in a spectral database of cells, which could then be used as a testbed for evaluating the performance of different multivariate analysis methods (Figure 1).

**Figure 1:**
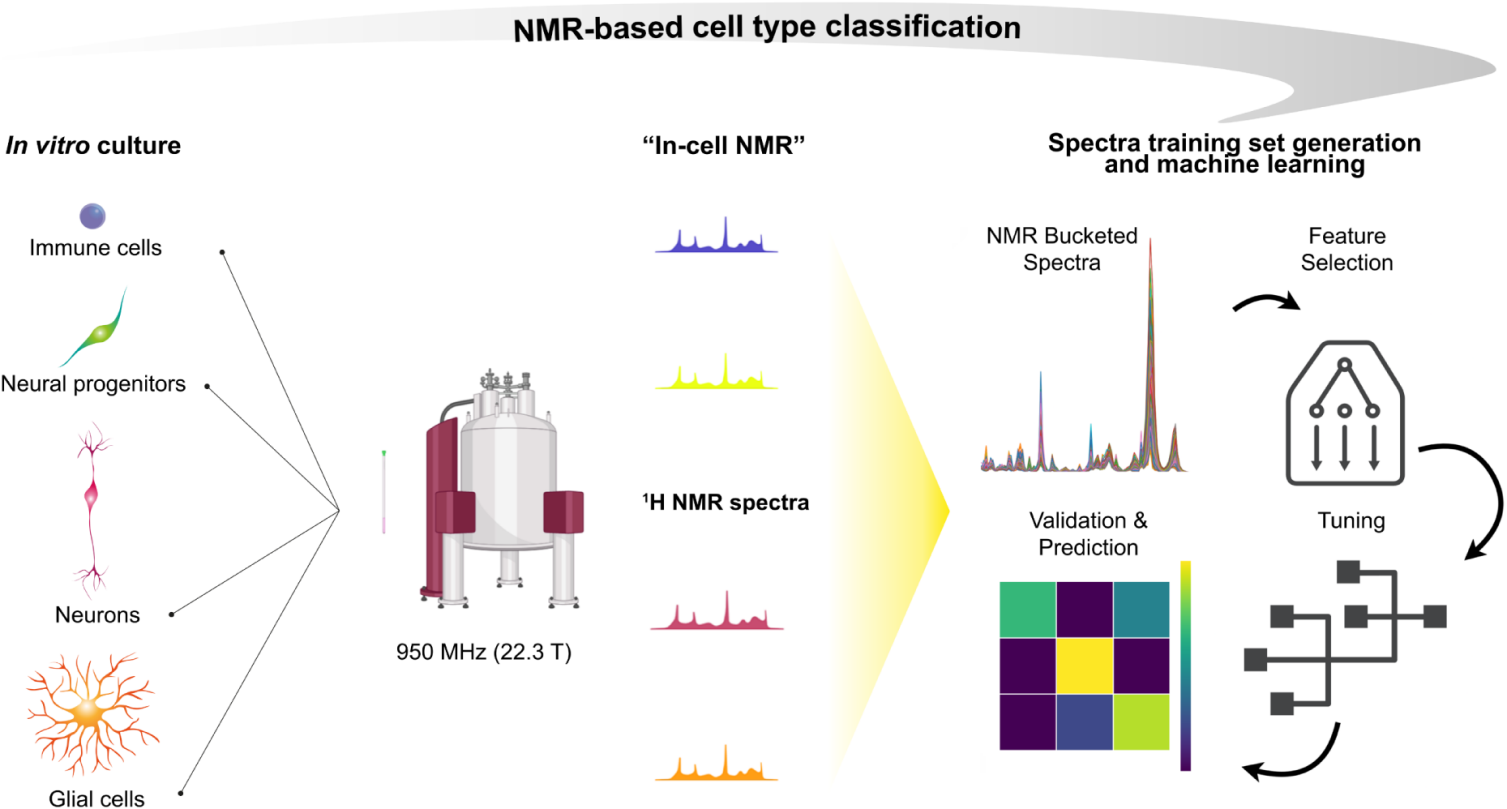
Overview of the workflow for sample preparation, NMR data collection, and multivariate analysis developed in this work.

Commercially available cell lines were used for HEK293T, HeLa, and Jurkat T samples, whereas samples of NPCs, neurons, and astrocytes were obtained with specific differentiation protocols using iPSCs. In particular, the NPCs employed in this study were derived from iPSCs via an optimized Dual SMAD inhibition protocol.^31,32^ NPCs were included to generate the differentiative trajectory in the spectral data set of neural progenitors, maturing neurons, and glial cells—underlining the translational potential of the approach. Furthermore, NPCs were differentiated toward neuronal fate by growth factor withdrawal and brain-derived neurotrophic factor (BDNF) administration^29^ (Figure S1) and were analyzed along the differentiation process across multiple time points, from D30 (day 30) to D90. We additionally generated astrocytes starting from iPSCs^33^ (Figure S2), which were driven toward a neural fate and then differentiated into astrocyte progenitor cells (APCs) and, subsequently, into mature astrocytes.

To maximize the intracell type spectral variability in the database, thereby improving the robustness of any subsequent analysis, samples of each cell type were collected at different numbers of passages or differentiative stages, and at different cell densities/confluencies. Two ^1^H NMR experiments were recorded on each cell sample: an excitation sculpting sequence, which includes resonances from all mobile components and was recorded with an intentionally short repetition time to maximize sensitivity per unit time, and a Carr–Purcell–Meiboom–Gill (CPMG) sequence, which filters out slow-tumbling molecules and contains signals only from small metabolites.^34,35^ The resulting database contains CPMG and excitation sculpting spectra recorded on a total of 174 samples of various cell types (Figures S3–S8). A tentative assignment of the most prominent spectral features in the CPMG spectra is shown in Figures S9 and S10.

### Classification of Four Different Cell Types from NMR Spectra

A supervised PLSDA model was trained on the ^1^H CPMG NMR spectra for cell line classification (Figures S3 and S4). As opposed to unsupervised methods such as Principal Component Analysis (PCA), which use the data covariance matrix to identify components that maximize the variance in the data set, PLSDA projects the spectra onto latent variables that maximize the separation between groups of data (classes) according to predefined labels (here, the different cell types). The separation of the classes projected in the latent space is then evaluated using SVM, which finds the optimal margins to separate each class, defining regions based on which newly projected samples can be assigned. The classification performance for the 4-class problem (HeLa, HEK, Jurkat, NPCs) was evaluated using the F_1_ score (i.e., the harmonic mean of precision and recall^36^) and resulted in a remarkably high average F_1_ score of 0.96 (Figure 2). A lower classification performance was obtained with the same model trained on the ^1^H excitation sculpting NMR spectra, suggesting that the slow-tumbling spectral components present in the latter type of spectra impair the PLSDA classification, especially between the HEK293T and HeLa cell classes (F_1_ score of 0.68, Figure S11).

**Figure 2:**
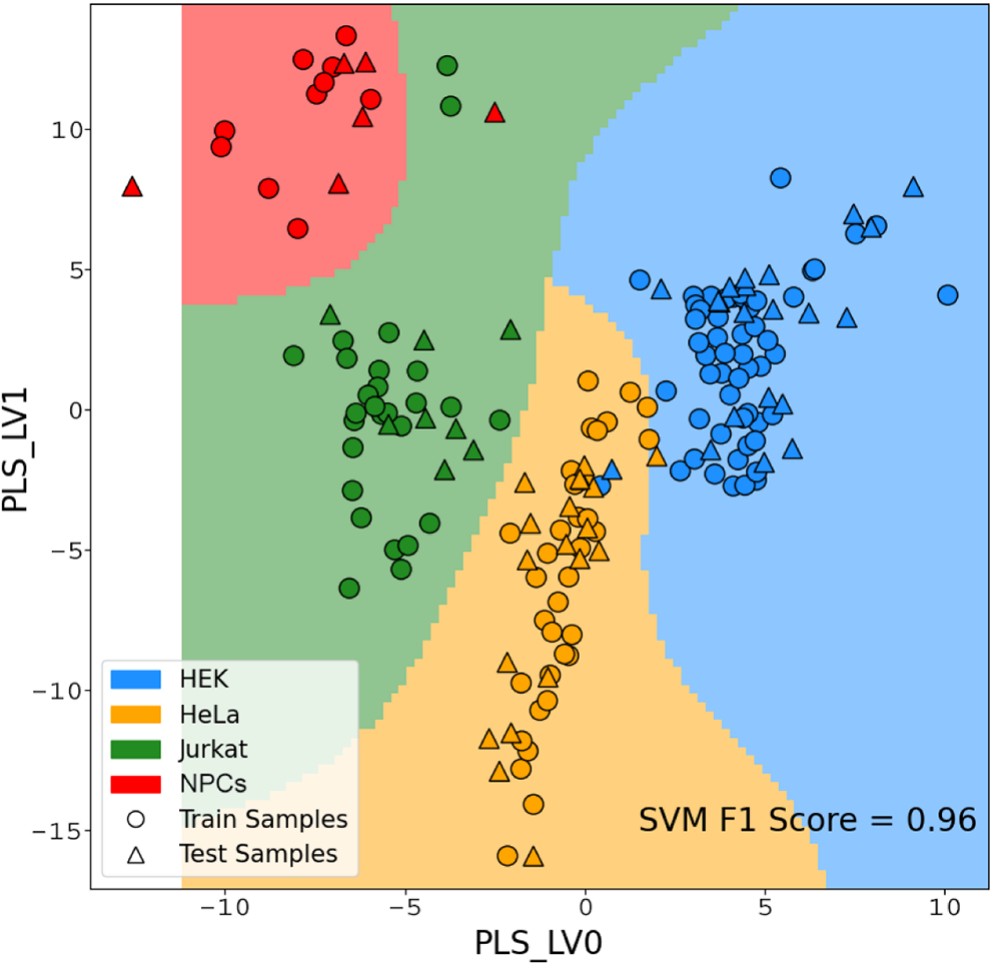
Projection of the first two components of the Partial Least Squares Discriminant Analysis (PLSDA), i.e., those which maximize the separation of different cell types, of the 4-class model trained on the ^1^H CPMG NMR spectra (see Figures S3 and S4), and classification margins as estimated by the Support Vector Machine (SVM). Training (circles) and validation (triangles) samples and SVM boundaries are colored based on the class: HEK (blue), HeLa (yellow), Jurkat (green), and NPCs (red).

### Separation of CNS Cell Types

The high intraclass variability observed for HeLa cells prompted us to test the ability of multivariate analysis to resolve cell phenotypes as they change over time. This ability is indeed relevant in the perspective of applying machine learning approaches to discriminate between healthy CNS components and malignancies. Indeed, cancer cells often exhibit metabolic NPC-like properties.^37,38^ For this reason, we sought to determine whether NPCs can be discriminated from differentiated neurons and glial cells. To this aim, we analyzed through PLSDA and PCA the samples of NPCs, both undifferentiated and undergoing differentiation toward early and late neurons, together with APCs and mature astrocytes derived from iPSCs (Figure S7). The PLS plot obtained from ^1^H CPMG NMR spectra shows that NPCs, as well as early and late neurons, APCs, and astrocytes, cluster in distinct regions (Figure 3A). Interestingly, PCA fails to separate all cell types but still separates NPCs/neurons from astrocytes, suggesting that the spectral differences among these cell types are such that even an unsupervised analysis can isolate them in distinct clusters (Figure 3B). A similar result was obtained from ^1^H excitation sculpting NMR spectra (Figures S8 and S12), although with worse separation between early and late neurons. Remarkably, in both PLS analyses, NPCs undergoing differentiation to neurons seem to follow a trajectory across the PLS plane, with early neurons clustering midway between NPCs and late neurons. Furthermore, the APCs-to-astrocytes trajectory is clearly separated from that of NPCs-to-neurons. Overall, this result highlights the potential of multivariate analysis to track changes in the phenotype of intact cells, as well as the benefits of high-field NMR in terms of sensitivity when analyzing small-size cell samples (1–2 million cells confined in 0.44 mm capillaries). The capability of discriminating undifferentiated cells from differentiated neurons and astrocytes is indeed promising from a clinical perspective, suggesting that multivariate analysis could allow MRSI-based virtual biopsies in the CNS disease evaluation.

**Figure 3:**
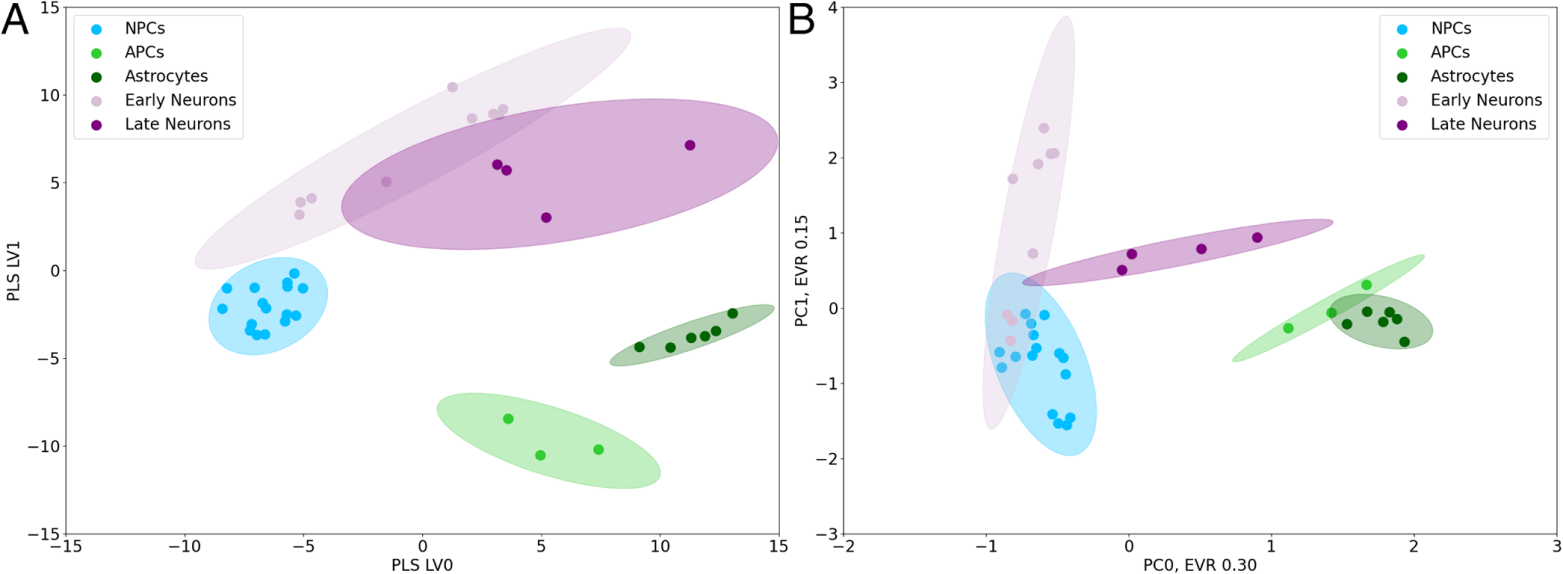
PLSDA (A) and PCA (B) analysis of ^1^H CPMG NMR spectra of a collection of CNS cell types. Samples are color-coded based on the stage of differentiation: neural progenitor cells (NPCs, light blue), early neurons (pink), late neurons (purple), astrocyte progenitor cells (APCs, light green), and mature astrocytes (dark green). 95% confidence ellipses calculated from the covariance matrix are shown. In part B, the explained variance ratio (EVR) for each PCA component is also shown.

### Classification of Cell Mixtures by Feature Selection

We then tested whether classification models trained on pure cell lines are capable of determining the composition of mixed samples, each containing two cell types. For this analysis, the PLSDA + SVM model was trained on 3 pure cell types (HEK293T, HeLa, and Jurkat; Figure 4A). The model was used to assign each mixed sample (Figures S13 and S14) to the following classes: HeLa + Jurkat, HEK + Jurkat, and HeLa + HEK (Figure 4B). The prediction was done by taking the two highest probability scores for the classes of pure cell types. The high intraclass variability of the HeLa samples in the PLSDA model negatively affected the classification performance: many mixed samples fell within the probability region of the HeLa class, resulting in poor classification performance, suggesting that PLSDA + SVM is unsuitable for determining the composition of mixed samples (Figure 4C).

**Figure 4:**
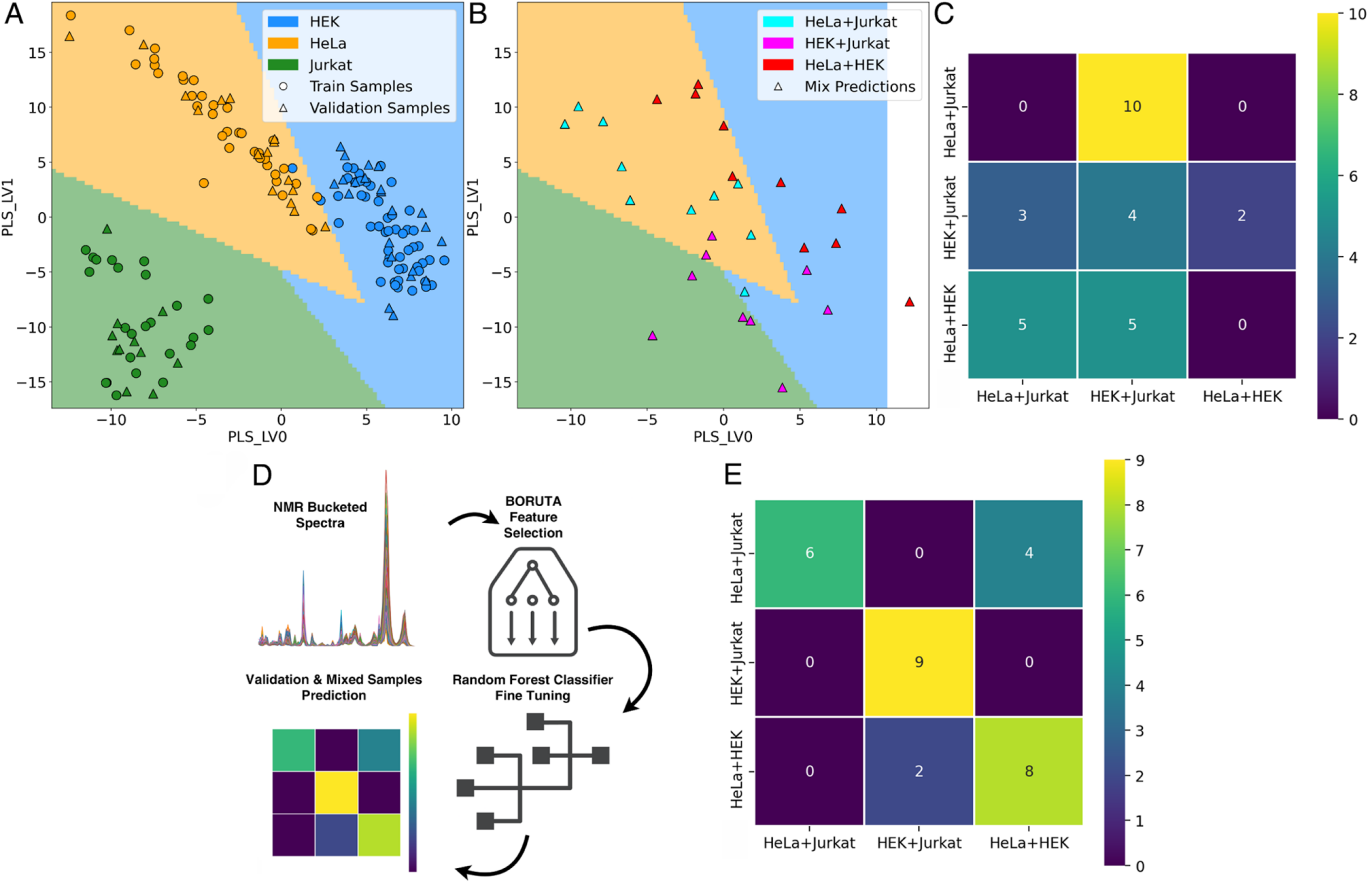
(A) Projection on the first two PLSDA components of the 3-class model trained on ^1^H CPMG NMR spectra (see Figures S3 and S4) and classification margins as estimated by the SVM. Training (circles) and validation (triangles) samples and SVM boundaries are colored based on the class: HEK (blue), HeLa (yellow), and Jurkat (green). (B) Mapping of the mixed samples (triangles, see ^1^H CPMG NMR spectra in Figure S13) on the 3-class PLSDA classifier. Samples are color-coded as follows: HeLa + Jurkat (cyan), HEK + Jurkat (purple), and HeLa + HEK (red). (C) Confusion matrix of the PLSDA + SVM model, trained on pure cell lines, used to predict mixed samples. This model fails the classification task, resulting in an F_1_ score of 0.05. (D) Schematic workflow of Boruta feature extraction followed by random forest classification (RFC). (E) Confusion matrix of the Boruta + RFC model. Out of 29 samples, only 6 are misclassified, resulting in an F_1_ score of 0.78.

While multivariate methods such as PCA and PLSDA are widely employed in metabolomics to disentangle the complexity of ^1^H NMR spectra, an alternative approach is based on the selection of original features to reduce the complexity of the data. One advantage of feature selection is that the spectral features discriminating the samples are identified directly, without the need to be reconstructed via inverse transformation, as required by multivariate methods. We therefore tested the Boruta algorithm for feature selection, followed by an RFC to select the top-performing features (Figure 4D). Feature selection for the 4-class problem resulted in 119 spectral features selected for training the RFC. The best model was then tested on the validation set, with a resulting performance of 1.00 (CPMG) and 0.96 (excitation sculpting) F_1_ score (Figure S15). For the prediction of mixed samples, the Boruta feature selection and RFC were optimized for the 3-class problem, resulting in 68 spectral features used for classification. The resulting optimized model, trained on pure cell lines, was then used to predict mixed samples. Also, in this case, mixed classes were assigned by taking the highest two predicted probability scores for the pure cell type classes. Strikingly, the Boruta + RFC approach resulted in a much higher classification performance (F_1_ score of 0.78, Figure 4E) compared to PLSDA + SVM (F_1_ score of 0.05, Figure 4C). The same comparative analysis was performed using ^1^H excitation sculpting NMR spectra (Figure S14) and resulted in an even higher performance of the Boruta + RFC approach (86%) compared to PLSDA + SVM (3%, Figure S16). Overall, although the high intraclass variability of HeLa samples also affected the Boruta + RFC approach (most misclassified samples contained HeLa cells), the latter proved to be much more robust than PLSDA + SVM in the classification of mixed samples using both CPMG and excitation sculpting ^1^H NMR spectra.

### Cell Type Classification at Lower Magnetic Fields

To test whether the above approaches can be translated to lower fields, approaching those currently reached in MRI instruments, we classified spectra acquired at 400 MHz (9.4 T) and 700 MHz (16.4 T) on samples of pure cell lines using a three-class PLSDA + SVM model trained with the data acquired at 950 MHz (22.3 T). To this aim, 21 samples of HEK293T, HeLa, and Jurkat were sequentially analyzed at 400, 700, and 950 MHz (Figures S17 and S18). Classification of these samples using the ^1^H CPMG NMR spectra recorded at the three fields resulted in F_1_ scores of 0.96 (950 MHz), 0.68 (700 MHz), and 0.96 (400 MHz) (Figure 5). Comparable performance was observed with the ^1^H excitation sculpting NMR spectra, resulting in F_1_ scores of 0.96 (950 MHz), 0.86 (700 MHz), and 0.91 (400 MHz) (Figure S19). Also, in this case, Boruta + RFC outperformed PLSDA + SVM, resulting in F_1_ scores of 1.00/0.96 (950 MHz), 0.95/0.95 (700 MHz), and 0.96/0.87 (400 MHz) for CPMG and excitation sculpting ^1^H NMR spectra, respectively (Figure S20). These results suggest that a model trained at high field is also capable of classifying data collected at lower fields, with a minimal decrease in performance. Visual inspection revealed that the ^1^H spectra collected at high field were not dramatically different from those collected at low field (Figures S17 and S18), consistent with the notion that the broadening of the spectral resonances from intact cells is dominated by the sample inhomogeneity, which scales linearly with the magnetic field, and therefore spectra at different fields show similar broadening once plotted on the ppm scale. Therefore, we expect that the challenge in classifying data collected at lower fields with models trained at high field will arise mainly from the lower S/N afforded by the low-field instruments (this is especially true for spatially resolved spectral data) and from phenomena that do not scale with the field and might alter the shape of the spectral features (scalar couplings, spin relaxation, and chemical exchange).

**Figure 5:**
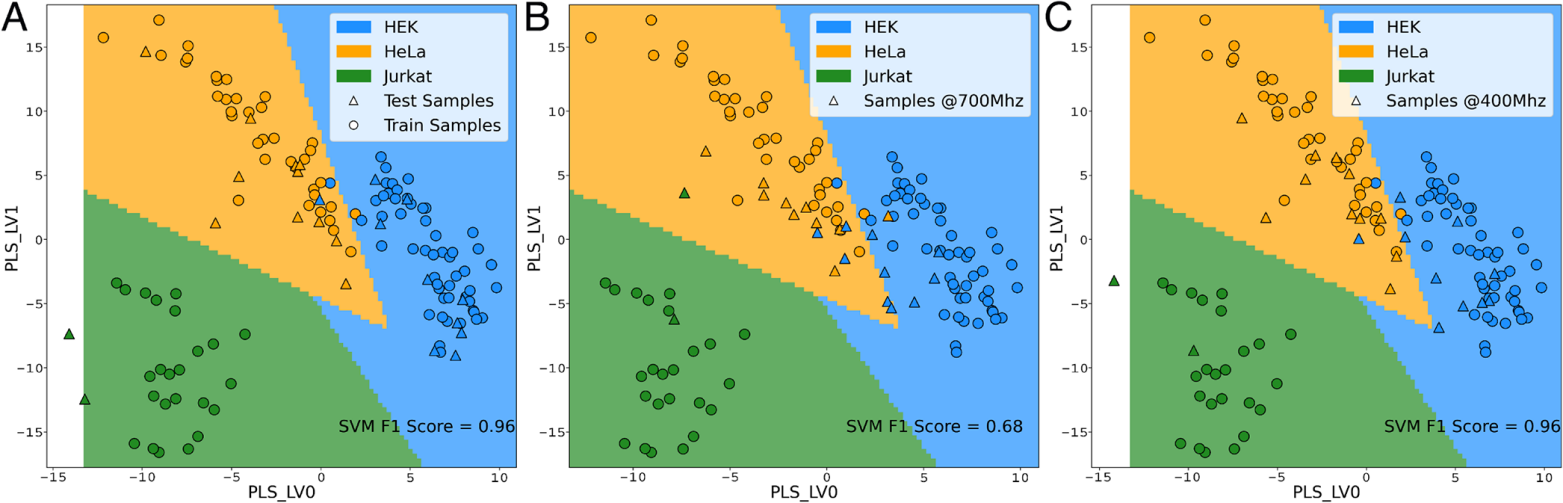
Classification of cell samples analyzed with ^1^H CPMG NMR (see Figure S17) at (A) 950 MHz (22.3 T), (B) 700 MHz (16.4 T), and (C) 400 MHz (9.4 T) based on the 3-class PLSDA + SVM model trained with data acquired at 950 MHz. Training (circles) and validation (triangles) samples and SVM boundaries are colored based on the class: HEK (blue), HeLa (yellow), and Jurkat (green).

### Performance Analysis and Interpretation of the Classification Models

As is common with machine learning methods, improved performance often comes with the risks of decreasing the method’s robustness and/or making the classification rules difficult to interpret. We therefore sought to evaluate the performance stability of the 4-class and 3-class PLSDA + SVM and Boruta + RFC classification models while simultaneously providing an interpretation in terms of the relevance of spectral features. For PLSDA, coefficient-coded loading plots provide an overview of which spectral features contribute to separating the cell types along each axis of the score plots (Figures S21 and S22). Variable Importance in Projection (VIP) scores rank the contribution of each feature to the predictive power of the classifier (Tables S1 and S2), while Jackknife analysis evaluates the stability of the highest-ranking features upon removal of subsets of samples (Tables S3 and S4). For the Boruta + RFC model, SHapley Additive exPlanations (SHAP) analysis ranks and clusters the features according to their overall impact on the model output. These results show that the classification performance is not driven by a very limited set of spectral features, consistent with a data structure that is inherently different from that of highly resolved metabolomics spectra of biofluids. With PLSDA, the numerous buckets with many close VIP values greater than 1 suggest that collinearity and redundancy play a big part when analyzing intact cells. Even though PLSDA is designed to handle collinearity, in this scenario most of the information driving the classification arise from small contributions spread across many correlated buckets, some of which likely arise from the same spectral envelope. Consistently, the analysis of sensitivity of PLSDA performance to the removal of top VIP-ranked buckets (Figures S23 and S24) shows that classification performances drop significantly only after the removal (in VIP score order) of roughly 23% of the total spectral features. Thus, PLSDA yields good performances with pure cell lines by retaining some of the redundant nature of the spectral envelopes. However, it fails to discriminate information in complex samples (Figure 4B) because it ultimately forces a representation through linear coefficients.

This fact becomes clearer through SHAP analysis of the Boruta + RFC model. First, the Boruta feature selection ensures that RFCs are trained only with spectral features that are strictly stable under random permutations. The SHAP results (Figures S25–S33) then show that the strength of the model comes from the fact that many trees reach the same decision through paths involving different sets of redundant features. The SHAP summaries, both global (Figures S25 and S30) and per-class (Figures S26–S29 and S31–S33, left panels), contain many of the features that presented high VIP scores, selectivity ratios, and good stability in the PLSDA models. However, computing the interaction among sets of features (Figures S26–S30 and S31–S33, right panels) unveils a picture of decision-making that mirrors the true nature of spectral envelopes more accurately. Indeed, most of the decision-making in the RFCs is determined by different trees of the random forest collapsing into paths of interacting/redundant features rather than a limited set of independent features giving strong contributions to classification. Thus, more general classifiers such as RFCs, which are not bound to linear assumptions for coefficient estimation, are capable of handling spectral information from broad, overlapping signals in complex samples, even when classifying cell mixtures or data recorded at different magnetic fields.

Among the most significant spectral features identified by both PLSDA and RFC, some could be tentatively assigned to known markers of cell metabolism (Figures S9 and S10). These include fatty acids, lactate, and choline (highest features in the loading plots, Figures S21 and S22), glutathione (8.3 ppm), creatine (3.1 ppm), and acetate (1.95 ppm). However, most of the significant features arose from minor peaks and overlapping regions that could not be assigned. This underscores the capability of PLSDA and RFC to select highly discriminating features not just from a few abundant metabolites but also from spectral features emerging from complex phenotypic patterns.

## Conclusions

In this work, live neural cell types, including NPCs, neurons, astrocyte progenitors, and mature astrocytes, as well as Jurkat lymphocytic cells (i.e., a cell type involved in most of brain infectious and autoimmune inflammatory lesions or in brain lymphomas), as well as immortalized cell lines (such as HEK) and cancerous HeLa cells, similar to those that can be found in primary non-CNS neoplasms that can invade the CNS as metastatic lesions, were analyzed by ultrahigh-field ^1^H NMR spectroscopy. These cell types provided relevant phenotypes in light of potential applications for evaluating CNS lesions through MRSI.

Even at these field strengths, the ^1^H NMR spectra of intact cells show extensive signal overlap and are therefore poorly resolved. Nonetheless, we showed that these cell samples are sufficiently diverse to disentangle their spectral fingerprint and allow correct cell type classification using an approach analogous to those employed in metabolomics by high-resolution NMR. Indeed, we showed that the sources of variability contained in the low-resolution ^1^H spectral data from living cells can be extracted by multivariate analysis, by training supervised classification models (SVM, random forest ensembles) with different data dimensionality reduction approaches (PLSDA, Boruta feature selection). This approach allowed separation with high accuracy of the spectral fingerprint specific to each different cell type examined. We then showed that this approach can separate neural progenitors (NPCs, APCs) from differentiated neurons and astrocytes, thus also demonstrating a remarkable capability to track developmental trajectory changes within each cell type. Furthermore, we showed that a model trained on samples of single cell types can provide a predictive spectral marker that can reliably recognize the correct phenotype in unknown samples obtained by mixing two different cell types. Even more importantly, we showed that spectral patterns identified at high field (22.3 T) are preserved at lower fields (16.4 T and even 9.4 T), and that a predictor marker built from high-field NMR spectra is applicable with reasonable accuracy to data sets recorded at lower magnetic fields. This finding suggests that similar results could be transferred to the magnetic fields currently available for MRSI in humans (7 T, 11 T). Notably, we found that Boruta feature selection coupled with random forest classification systematically outperformed PLSDA + SVM, especially in the classification of mixed samples, suggesting that the former algorithm could also find broader adoption in metabolic fingerprinting by high-resolution NMR to tackle more challenging analyses.

In conclusion, while this work does not yet show the application of machine learning analysis to *in vivo* MRSI data, it provides the proof of principle that poorly resolved spectral features extracted by machine learning can be exploited to classify cells based on their phenotypes with high accuracy. In this respect, we predict that the lower sensitivity afforded by MRSI will be the major challenge to overcome, rather than spectral resolution. Nevertheless, these results suggest that in the future, if trained on brain tissue samples or *in vivo* MRSI data, machine learning may be able to provide spectral MRSI markers good enough to allow *in vivo* the identification of cell type content within CNS lesions with sufficient accuracy to allow classification of these lesions according to their pathogenic nature. We envision that in the future this approach could contribute to overcome the current limitations of MRSI, implementing ^1^H NMR spectral classification as an alternative to the most invasive of the biopsies, the CNS one, thus developing a noninvasive tool accurate enough for the *in vivo* differential diagnosis of many CNS lesions. This “virtual biopsy” would potentially spare a large number of real invasive biopsies and would make diagnosis feasible in hard-to-access CNS sites.

## Experimental Section

### Cell Culture Maintenance

HeLa (obtained from the Swiss Institute for Experimental Cancer Research), HEK293T (American Type Culture Collection, ATCC CRL-3216), and Jurkat T lymphocytes (Interlab Cell Line Collection, ICLC HTL01002) were grown following the reagents and protocols recommended by the ATCC. HeLa and HEK293T cells were maintained in high-glucose Dulbecco’s modified Eagle medium (DMEM, Gibco) supplemented with l-glutamine (Gibco), antibiotics (penicillin and streptomycin, Gibco), and 10% fetal bovine serum (FBS, Gibco) in uncoated T75 plastic flasks and incubated at 37 °C, 5% CO_2_ in a humidified atmosphere. Both cell cultures were passaged by seeding at a 1/10 dilution twice a week. For NMR analysis, additional T75 flasks were seeded at the same density and collected at different time points postseeding (from 1 to 4 days). As each cell line has its characteristic growth curve and cell density is not easily measured in a quantitative way, a time parameter related to the cell density, “days after passage”, was used internally. Jurkat T lymphocytes were maintained in RPMI 1640 medium (Gibco) supplemented with MEM nonessential amino acids (Gibco), sodium pyruvate (Gibco), antibiotics, and 10% FBS (Gibco) in uncoated T75 plastic flasks and incubated at 37 °C, 5% CO_2_ in a humidified atmosphere. Cells were passaged by seeding 1 × 10^6^ viable cells (as determined by Trypan Blue staining) in a new T75 flask twice a week. HSB 311 iPSCs #1—employed for the generation of NPCs, neurons, and astrocytes—were obtained from skin fibroblasts through episomal reprogramming as previously described^39,40^ and maintained in Stem Flex Basal medium (Thermo Fisher Scientific, #A3349201) on Matrigel-coated culture plates (1:60, Corning, #356234). iPSCs were passaged every 5–6 days. Briefly, cells were incubated for 3 min at 37 °C with 0.5 mM EDTA to induce colony detachment. Following this, EDTA was removed, and culture dishes were rinsed with Stem Flex to collect cells in small clumps. Finally, iPSCs were plated at a 1:6–1:8 ratio in a new culture vessel.

### Generation of NPCs, Neurons, and Astrocytes

The NPCs used in this study have the identity of neuroepithelial stem (NES) cells and were derived as previously reported.^31,32^ Briefly, healthy iPSCs were plated at high density (2 × 10^5^/cm^2^) onto Matrigel-coated plates for neural induction. This phase foresees employing neural induction media (DMEM-F12 (Thermo Fisher Scientific, #11-330-057)/Neurobasal (Thermo Fisher Scientific, #21103049) mixture 1:1, 1% N2 (Thermo Fisher Scientific, #17502001), 2% B27 (Thermo Fisher Scientific, #17504-044), 20 μg/mL insulin (Sigma-Aldrich, #I9278), 1% MEM-nonessential amino acids (Thermo Fisher Scientific, #11140050), 1% l-glutamine (Thermo Fisher Scientific, #25030024), 0.1% 2-mercaptoethanol (Thermo Fisher Scientific, #21985023))—to be replaced daily—for 12 days, containing Dual SMAD inhibition factors. Indeed, by providing small molecules such as SB431542 (10 μM, TargetMol, #T1726), LDN193189 (100 nM, Stem Cell Technologies, #72144), and XAV939 (2 μM, Stem Cell Technologies, #72674), the TGFβ and BMP pathways are blocked, and proneural genes are activated. At the end of the neural induction phase, the obtained neuroectodermal cells were replated in bulk at high density (2 × 10^5^/cm^2^) in NES medium (DMEM-F12, 1% N2, 0.1% B27, 1.6 g/L glucose (Sigma-Aldrich, #RDD016), 20 μg/mL insulin), supplemented with 20 ng/mL FGF2 (Peprotech, #100-18B), 20 ng/mL EGF (Peprotech, #315-09), and 5 ng/mL BDNF (Peprotech, #450-02). Under these culture conditions, NPCs can be maintained in self-renewal and can be long-term expanded.^22^ Mature neurons with a neocortical identity were differentiated from NPCs. Following the withdrawal of growth factors and the administration of 30 ng/mL BDNF in neuronal differentiation medium (DMEM-F12/Neurobasal mixture 1:1, 0.5% N2, 1% B27, 10 μg/mL insulin, 1% l-glutamine), NPCs undergo terminal differentiation.^22^ Early (∼30 days), mid (∼60 days), and late (∼90 days) time points were considered for NMR analysis.

For astrocyte generation, iPSCs were differentiated as described previously.^33^ Briefly, iPSCs were dissociated into single cells and plated at a density of 5 × 10^4^ cells/cm^2^ on poly-d-lysin (PDL) (Sigma-Aldrich, #P6407-5MG)/laminin-coated dishes (Sigma-Aldrich, #L2020) in N2B27 medium supplemented with 4 ng/mL FGF2, 500 ng/mL LDN193189, 20 μM SB431542, and 10 μM Y-27632. After 10 days, NPCs were collected and maintained for 8 passages on PDL/laminin-coated dishes in N2B27 medium supplemented with 10 ng/mL FGF2, 10 ng/mL EGF, and 20 ng/mL BDNF. At passage 8, NPCs were plated on Vitronectin-treated plastic (Thermo Fisher Scientific, #A14700) and exposed to astrocyte-committing media (Neurobasal—DMEM/F12 1:1 + 0.5% N2, 1% B27, 20 ng/mL FGF2, and 20 ng/mL EGF) for 25 days to induce Astrocyte Progenitor Cell (APC) commitment. Finally, APCs were seeded (2 × 10^4^ cells/cm^2^) on Vitronectin plates in astrocyte maturation media (DMEM/F12 + 1% N2, 10 ng/mL CNTF (Peprotech, #450-13-100UG), and 10 ng/mL BMP4 (Peprotech, #120-05ET)). Astrocytes were then cultured for a further 50 days (AMM).

All iPSC and NPC work was performed according to NIH guidelines for the acquisition and distribution of human tissue for biomedical research purposes and with approval by the Human Investigation Committees and Institutional Ethics Committees of each institute from which samples were obtained. Final approval from the Committee on Bioethics of the University of Pisa was obtained (Review No. 29/2020).

### Preparation of NMR Samples

Sample preparation for NMR analysis was performed by adapting an existing protocol.^41^ HEK293T, HeLa, and Jurkat cells from T75 flasks were detached with Trypsin 0.25%–EDTA, inactivated with DMEM + 10% FBS, spun down at 800 g for 5 min, washed once with PBS and another time with PBS buffer + 5% D_2_O, resuspended with 180 μL of the same buffer, and placed in a 3 mm Shigemi tube for NMR analysis, forming a soft pellet at the bottom of the tube. Cell numbers varied depending on confluency on the day of collection. Therefore, cells from either 1, 2, or 3 T75 flasks were pooled together in order to properly fill the NMR tube (∼3 × 10^7^ cells per NMR sample). NPCs, neurons, and astrocytes (∼1–2 × 10^6^ cells for each NMR sample) were detached with Trypsin 0.25%–EDTA (NPCs and astrocytes) or Accutase (neurons), inactivated with 4 volumes of PBS + 10% FBS or 4 volumes of PBS, respectively, spun down at 200 g for 3 min, washed once with PBS, and gently centrifuged. After PBS removal, cells were resuspended in 5–10 μL of PBS + 5% D_2_O and loaded into a glass capillary (Hilgenberg GmbH, borosilicate glass, 10 mm length, 0.44 mm i.d.) by capillarity. The capillary was flame-sealed on the clean end, placed in an empty 3 mm tube for NMR analysis, and spun down gently to form a soft pellet at the bottom of the capillary.

Samples of mixed cell lines were obtained by mixing suspensions of two different cell lines, prepared as described above, at varying percentages. Three cell lines were used: HeLa, HEK, and Jurkat, thus producing three classes of samples: HeLa + Jurkat, HEK + Jurkat, and HeLa + HEK.

### NMR Data Acquisition and Processing

Unless otherwise specified, all the NMR spectra were collected at 310 K (37 °C) with a Bruker Avance III HD 950 MHz (22.3 T) spectrometer equipped with a TCI Cryoprobe. 1D ^1^H excitation sculpting spectra were recorded with the *zgesgp* pulse sequence from the Bruker library with a 20 ppm spectral window, 32k points, 865 ms acquisition time, 4 dummy scans, 128 scans, and a 1 s interscan delay (total acquisition time of ∼4 min). Carr–Purcell–Meiboom–Gill (CPMG) spectra were recorded with the *cpmgpr1d* pulse sequence from the Bruker library, with a 20 ppm spectral window, 112k points, 3.03 s acquisition time, 4 dummy scans, 128 scans, and a 4 s interscan delay (total acquisition time of ∼16 min). 1D NOESY spectra, which are typically employed to enhance signals of slow-tumbling molecules,^42^ were not recorded due to time constraints required for ensuring cell sample stability. The spectra were processed with 5 Hz exponential line broadening, zero-filled, phase-corrected, and referenced to a signal at 6.01 ppm by using Topspin 3.6. This signal was tentatively assigned to either uridine monophosphate (UMP) or a uridine diphosphate (UDP) sugar using the Human Metabolome Database (HMDB).^43^ It was chosen because it was well-resolved in all the spectra and was not affected by water suppression artifacts. Uninteresting zones of the spectra were clipped out (over 8.6 ppm and under −0.1 ppm, as well as the water signal region between 5.1 and 4.6 ppm). The spectra were uniformly divided in 0.04 ppm-wide buckets. This bucket size was chosen over smaller sizes because it is comparable to the line width of the sharpest peaks in the cell spectra and allows reducing the total number of features to avoid overfitting, which is a known issue in PLSDA when the features/samples ratio is very high.^44^ Normalization was carried out by Probabilistic Quotient Normalization (PQN),^45^ using the median spectrum as the reference. After the spectra were clipped and bucketed, a total of 217 spectral features were retained for the analyses. Tentative assignment of the most prominent or well-resolved features in the CPMG spectra was obtained by integrating chemical shift information from Chenomx NMR Suite 8.3 (Chenomx Inc., Canada), the BMRB,^46^ the HMDB,^43^ and metabolite assignments taken from the literature.^47,48^

### Multivariate Analysis and Machine Learning

An automated framework for loading and processing the spectra was developed by using Python 3.8. Two different approaches were explored. In the first one, a dimensionality reduction of the spectra based on PLSDA was employed, followed by the training of a SVM on the dimensionally reduced data set.^26^ PLSDA is capable of compressing spectral features into a space where the variance of the data related to the classification task is maximized. More specifically, PLSDA latent variable coefficients are computed according to the univariate effect of each original spectral feature on the class prediction; each newly computed latent variable is then iteratively orthogonalized with respect to those already computed to ensure projections in a space where the correlation with the output is maximized and the collinearity between starting features is minimized. In this study, PLSDA was used to reduce the dimensionality of the spectral data set and project the samples into the space where the separation of different cell types is optimal. A SVM was then trained on the transformed data to assess the best separation boundaries for the classes in the dimensionally reduced projection space. The goal of the SVM is to find the best partition of the lower dimensional space for classification by assessing boundaries of decision that delimit each class probability region. Size and shape of the margins are optimized through a stochastic grid search of SVM hyperparameters. In the 4-class scenario, the SVM model converged to a radial basis function kernel to optimally separate classes, whereas in the 3-class scenario, it automatically converged to a linear kernel function. The model was trained through a stochastic grid search to optimize its hyperparameters, especially the kernel function that determines the shape of the decision boundaries. 70% of randomly selected samples were used as training, while the remaining 30% was used as validation. In this way, the model is flexible, with respect to classification task complexity. Results are shown for a 3-class and a 4-class classification problem, demonstrating that this type of framework can adapt as new classes of samples are acquired and added to the model. The second approach is based on Random Forest Ensemble, for both feature selection and classification. Feature selection was performed with the Boruta algorithm,^27^ an all-relevant feature selection method, which aims to find all features carrying information for prediction, rather than extracting a subset of features on which some classifier has minimal error. Boruta provides a solid choice for feature selection as the only parameter that controls the strictness of the selection is a threshold on the percentile of the distribution of the successes over tries of each feature against its random permutation. The best features selected by the algorithm were then fed to a random forest classifier by selecting those features that reliably perform better in the classification task with respect to their random permutations (shadow features). The filtered spectral features (nominally, the spectral buckets) were then passed to the real RFC, consisting of a random forest ensemble where concurrent trees are trained against each other to explore subsets of features and gather the top performing. The RFC was optimized during training through a stochastic grid search on the classifier hyperparameters of the ensemble (number of concurrent trees, maximum depth of estimators, minimum impurity decrease in a split, etc.). The model was then validated on a blind subset of data using a stratified k-fold cross-validation to avoid overfitting. 70% of randomly selected samples were used as training, while the remaining 30% were used as validation. For the RFC classifier at lower fields, the above training set (70% of the samples at 950 MHz) was validated on 100% of the samples at 700 and 400 MHz. A ranking of the features, based on Gini impurity, is also made available after the convergence to the best model, allowing the comparison of the most influential features among classes. Figure S34 shows a visualization of the decision path of one of the trees in the resulting best model. The data were standard-scaled for both models: for PLSDA, this is required to be within the assumptions of the methods (0 means and unit variances for the features); for the RFC, standard scaling helps reduce biases when ranking features using the Gini impurity. Both models were evaluated on a 5-fold cross-validation of the validation set using the F_1_ score, which is the harmonic mean of precision and recall, instead of accuracy,^36^ as it is less biased than accuracy in multiclass scenarios and when dealing with classes with different sample sizes.

### PLSDA Evaluation and Interpretation

To ensure a complete evaluation of the PLSDA models employed, we implemented a dedicated library in Python 3.8, based on SIMCA-P metrics and tools, which are widely recognized in chemometrics and multivariate analysis.^49^ The library is completely integrated with the SciKit-Learn library, which is the standard for a wide array of machine learning algorithms in Python, to ensure reproducibility and compatibility. The PLSDA models trained for the 3-class and 4-class applications were evaluated using the following:

*Permutation Testing for Cross-Validation (CV) Performance:* We assessed the robustness of CV scores, F_1_ scores for the present case, on random permutations of class labels. CV scores of the true classification tasks are tested against the null hypothesis that class labels have no relation to the data. The estimated *p*-value is indicative of the model performing with a certain CV in a way that is unlikely under class label randomness. Permutation testing resulted in F_1_ scores of 0.9866 and 0.9978 for a 5-fold cross-validation during training for the 4-class and 3-class PLSDA, respectively, both with a *p*-value of 0.0009 from testing against the null hypothesis.

*Variable Importance in Projection (VIP) Scores Computation:* VIP gives a score for each feature that summarizes how strongly that variable contributes (via the PLS weights) to the latent components that explain variation in Y (the output matrix, the targets).^49^ Thus, it is a measure of overall discrimination across all classes of a feature.

*Selectivity Ratios (SR):* SR is defined as the ratio of predictive variance to orthogonal variance for a certain feature.^49^ In a multiclass scenario, it can be used to rank variables by how selectively they support the discriminant direction associated with a certain class regression vector. In other words, it is a measure of how strongly a feature supports discrimination for a specific class.

*Jackknife Confidence Intervals (CI) for Regression Coefficients:* Stability analysis of regression coefficients for each class, under data set resampling.^49^ The model is refitted by removing stratified subsets of samples and evaluating the stability in regression coefficient estimates. In the present study, coefficient standard error (SE) and 95% CIs are estimated with a 5-fold stratified refitting (to ensure that enough samples for each class are represented in each fold). Low SE, narrow CI, and consistent sign (lower and upper CI bounds do not cross 0) define a coefficient that is stable under resampling.

### RFC Interpretation Using SHapley Additive exPlanations (SHAP)

To gain insight on how the RFC model performs classification tasks and understand the structure of information in spectral envelopes in this study, we resorted to the SHAP approach.^50^ SHAP is a paradigm for explaining how individual predictions are made by a machine learning model that basically decomposes a prediction into a sum of contributions from each of the model’s input features. An overview of the approach, together with the documentation for the Python library employed here, is provided by Lundberg et al.^51^

The Supporting Information reports the results of applying a TreeExplainer^52^ to obtain a global summary of SHAP values and beeswarm plots of SHAP values per class for each classification task (Figures S25–S33). This analysis highlights how spectral features contribute to decision paths in the RFC models and provides insight on spectral buckets and spectral feature value characterizing each class. Furthermore, we employed a model-agnostic Permutation Explainer to evaluate how the spectral data structure, in terms of feature redundancy and interactions, defines class decisions. In this way, we can peer into the nested data structure underlying broad overlapping signals by switching from Shapley values (which result from treating each feature independently) to Owen values through a recursive application of Shapley values to groups of features.^50,51^

## Supplementary Material



## Data Availability

All data are available in the main text or the Supporting Information. Raw NMR data are openly available on Zenodo at: 10.5281/zenodo.15858420. Code is available upon request.

## Notes

Supplementary Figures: Fluorescence images of iPSC cells, neurons, and astrocytes; ^1^H NMR spectra of all samples of pure cell lines; assignment of spectral features; 4-class PLSDA of the excitation sculpting NMR spectra; PLSDA and PCA plots of excitation sculpting NMR spectra of CNS-related cell types; ^1^H NMR spectra of cell mixtures; performance of the 4-class Boruta+RFC model; 3-class PLSDA of the excitation sculpting NMR spectra and performance of cell mixture classification; ^1^H NMR spectra recorded at different magnetic fields; classification performance of excitation sculpting NMR spectra at different fields; PLSDA coefficient-coded loading plots; plots of PLSDA performance sensitivity; SHAP summary and per-class plots of Boruta+RFC models; example of the decision path of a tree inside the random forest ensemble; Supplementary Tables : VIP scores of top 20 PLSDA features in the 4-class and 3-class problems; per-class Jackknife confidence intervals (PDF)
