# Supplementary material for: Phenotype Classification of Intact Cells by NMR Spectroscopy through Machine Learning Approaches
Source: J Am Chem Soc. 2026 Apr 22;148(17):17920–30. doi: 10.1021/jacs.6c01100 (PMC13154173; doi:10.1021/jacs.6c01100)
Supplement: Supplementary file 1 [file ja6c01100_si_001.pdf]

Supporting Information

# Phenotype classification of intact cells by NMR spectroscopy through machine learning approaches

Carlo Mengucci[1,†], Claudia Dell'Amico[2,3†], Simona Del Giudice[4], Letizia Barbieri[5], Alice Mariottini[6,7], Marco Onorati[2], Luca Massacesi[6,7], Enrico Luchinat[4,8*], Lucia Banci[4,5,8*]

[1] Department of Agri-Food Science and Technology, University of Bologna, Cesena 47521, Italy;
[2] Department of Biology, University of Pisa, Pisa 56126, Italy;
[3] Department of Clinical and Experimental Medicine, University of Pisa, Pisa 56126, Italy;
[4] Magnetic Resonance Center – CERM, University of Florence, Sesto Fiorentino 50019, Italy;
[5] Interuniversity Consortium for Magnetic Resonance of Metalloproteins – CIRMMP, Sesto Fiorentino 50019, Italy;
[6] Department of Neurosciences, Psychology, Drug research and Child Health, University of Florence, Florence 50139, Italy;
[7] Department of Emergency Neurology, Careggi University Hospital, Florence 50139, Italy.
[8] Department of Chemistry "Ugo Schiff", University of Florence, Sesto Fiorentino 50019, Italy;
† These authors contributed equally to the work
* Email: eluchinat@cerm.unifi.it; banci@cerm.unifi.it

**Table of Contents** **Page**

**1. Supplementary Figures** **2**
Figures S1-S34 2-32
**2. Supplementary Tables** **33**
Tables S1-S4 33-38

## 1. Supplementary Figures

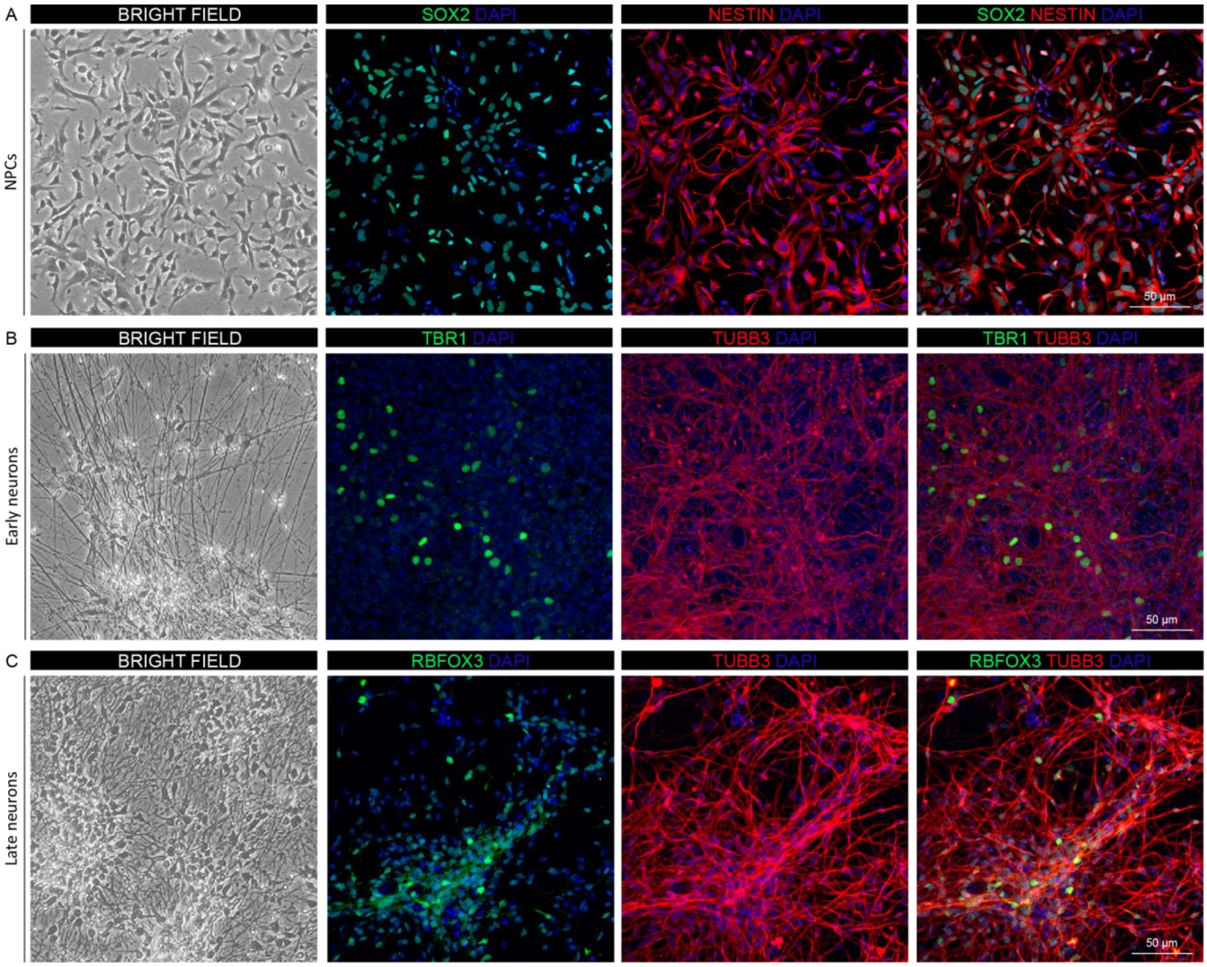


**Figure S1.** Characterization of iPSC-NES cells (i.e., NPCs) and NES-derived neurons. (A) Representative bright field and confocal immunofluorescence images of NPCs, positive for the neural stem/progenitor makers cell SOX2 (SRY-box transcription factor 2) and Nestin (intermediate filament). (B) Representative bright field and confocal immunofluorescence images of early-born neurons positive for the (T-box brain transcription factor 1, cortical layer 6) and for TUBB3 (β-III tubulin). (C) Representative bright field and confocal immunofluorescence images of late-born neurons positive for the mature pan-neuronal marker RBFOX3 (RNA Binding Fox-1 Homolog 3) and TUBB3. Nuclei are counterstained with DAPI. Scale bars: 50 µm.

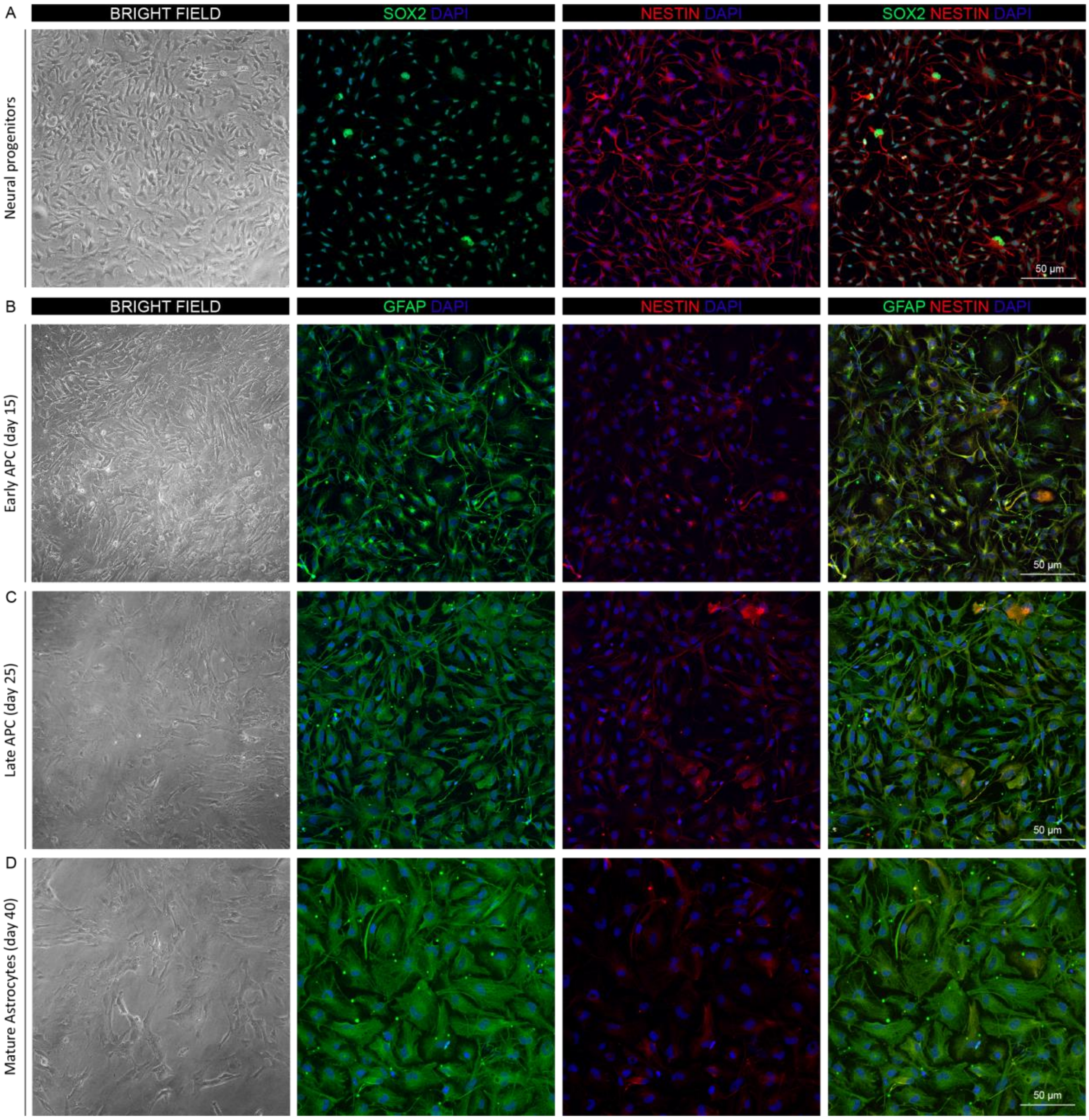


**Figure S2.** Characterization of the iPSC-derived astrocytes. (A) Representative bright field and confocal immunofluorescence images of neural progenitors positive for the SOX2 and Nestin. (B) Representative bright field and confocal immunofluorescence images of astrocyte progenitor cells (APC) expressing the glial marker GFAP (Glial fibrillar acidic protein), while Nestin expression is reduced. (C) Representative bright field and confocal immunofluorescence images of late APCs showing positivity for GFAP. (D) Representative bright field and confocal immunofluorescence images of mature astrocytes showing homogeneous GFAP expression. Nestin is barely detectable. Nuclei are counterstained with DAPI. Scale bars: 50 µm.

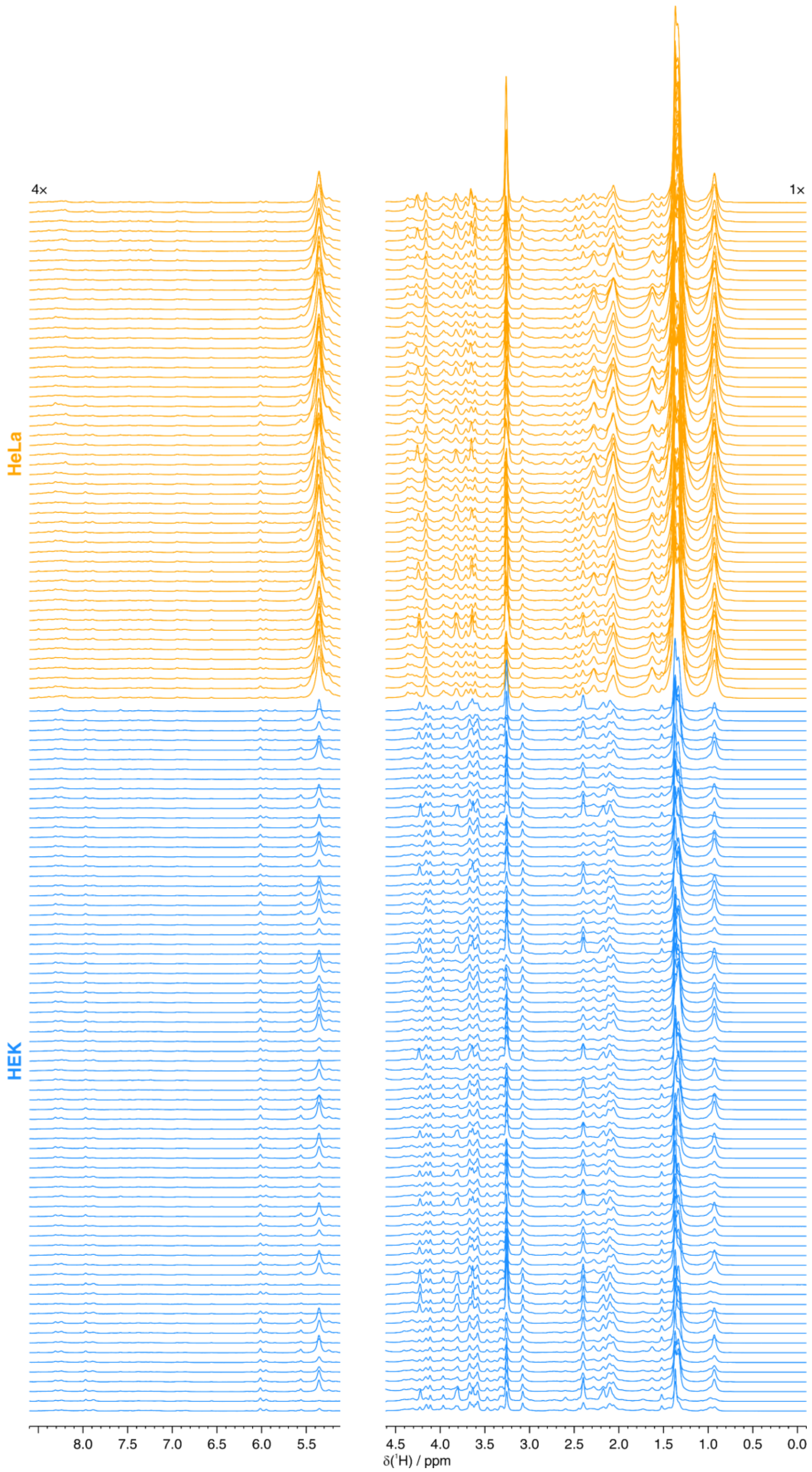


**Figure S3.** $^{1}$H CPMG NMR spectra collected at 950 MHz (22.3 T) on samples of HEK293T (blue) and HeLa (yellow) cells. The aromatic region (5.1-8.6 ppm) is scaled 4x for clarity.

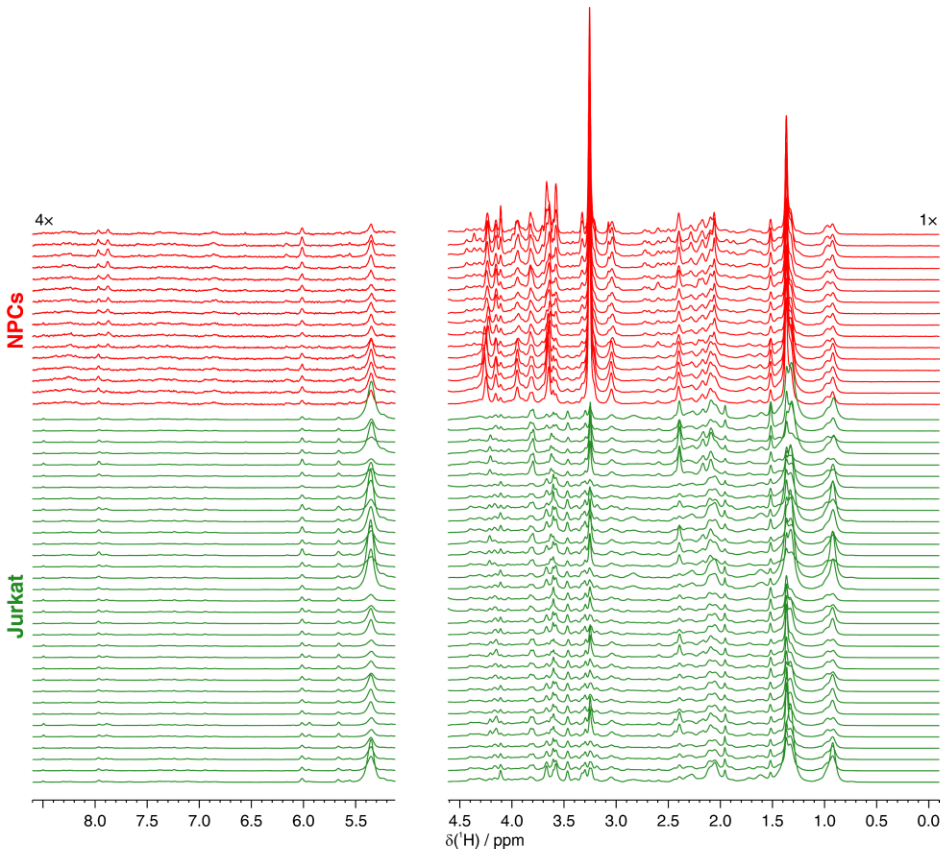


**Figure S4.** $^{1}$H CPMG NMR spectra collected at 950 MHz (22.3 T) on samples of Jurkat T lymphocytes (green) and NPCs (red). The aromatic region (5.1-8.6 ppm) is scaled 4x for clarity.

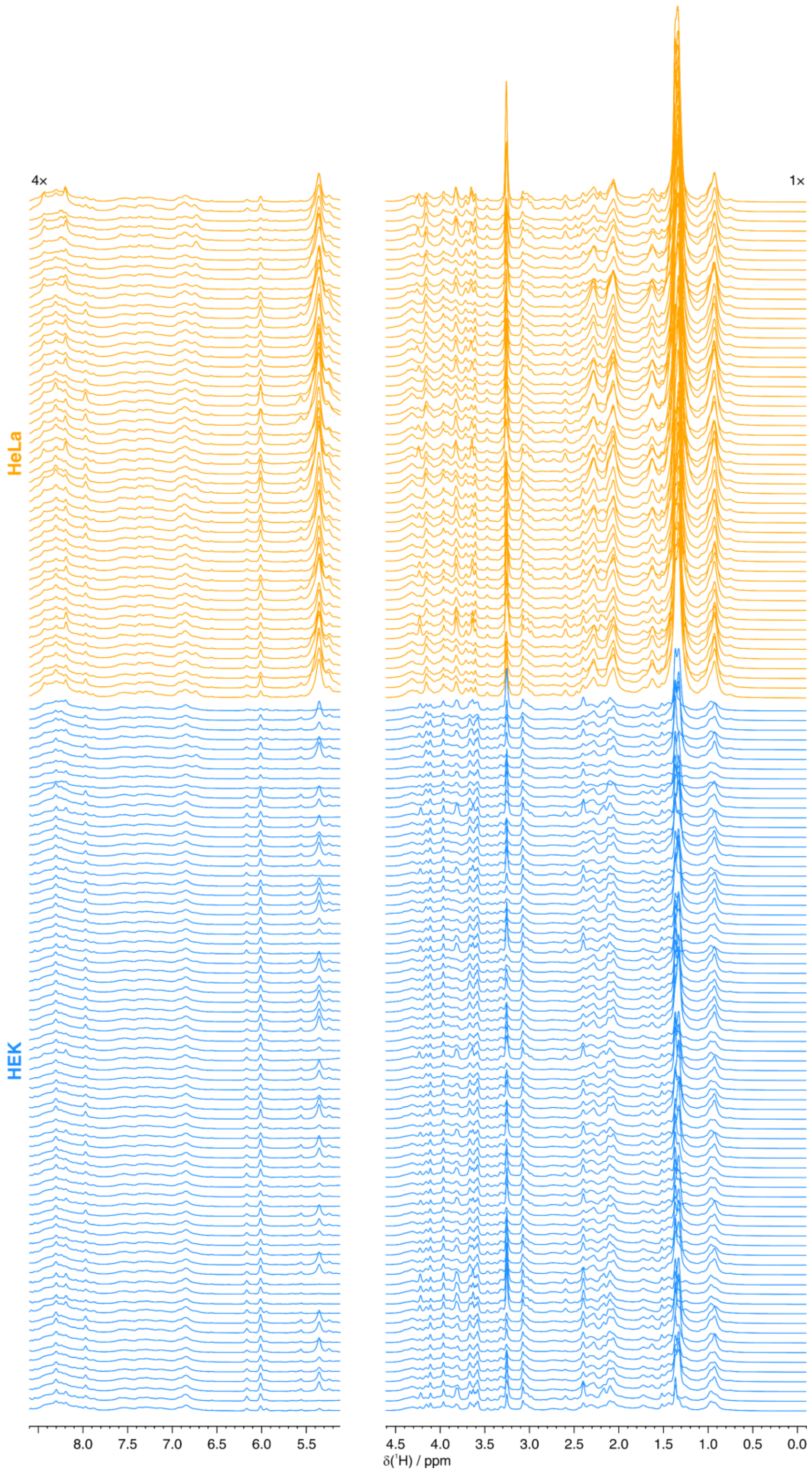


**Figure S5.** $^{1}$H excitation sculpting NMR spectra collected at 950 MHz (22.3 T) on samples of HEK293T (blue) and HeLa (yellow) cells. The aromatic region (5.1-8.6 ppm) is scaled 4x for clarity.

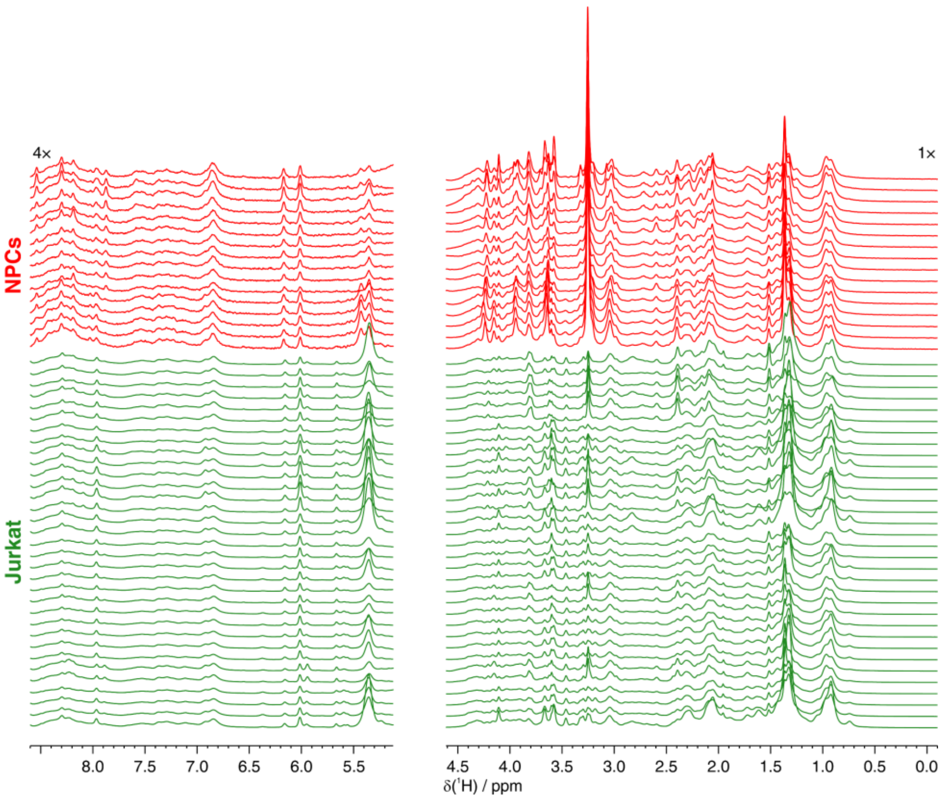


**Figure S6.** $^{1}$H excitation sculpting NMR spectra collected at 950 MHz (22.3 T) on samples of Jurkat T lymphocytes (green) and NPCs (red). The aromatic region (5.1-8.6 ppm) is scaled 4x for clarity.

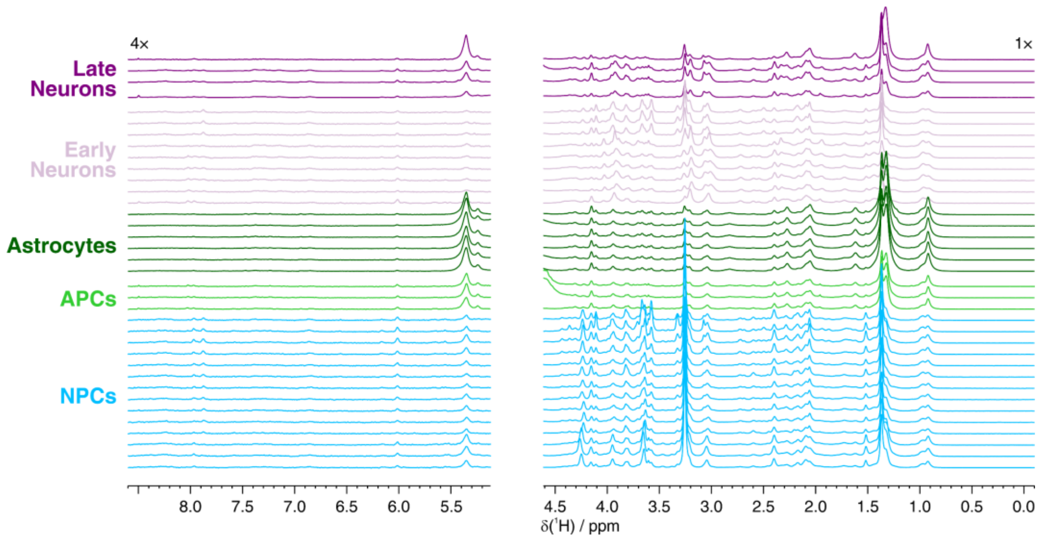


**Figure S7.** $^{1}H$ CPMG NMR spectra collected at 950 MHz (22.3 T) on samples of neural progenitor cells (NPCs, light blue), astrocyte progenitor cells (APCs, light green), astrocytes (dark green), early neurons (pink), and late neurons (purple). The aromatic region (5.1-8.6 ppm) is scaled 4x for clarity.

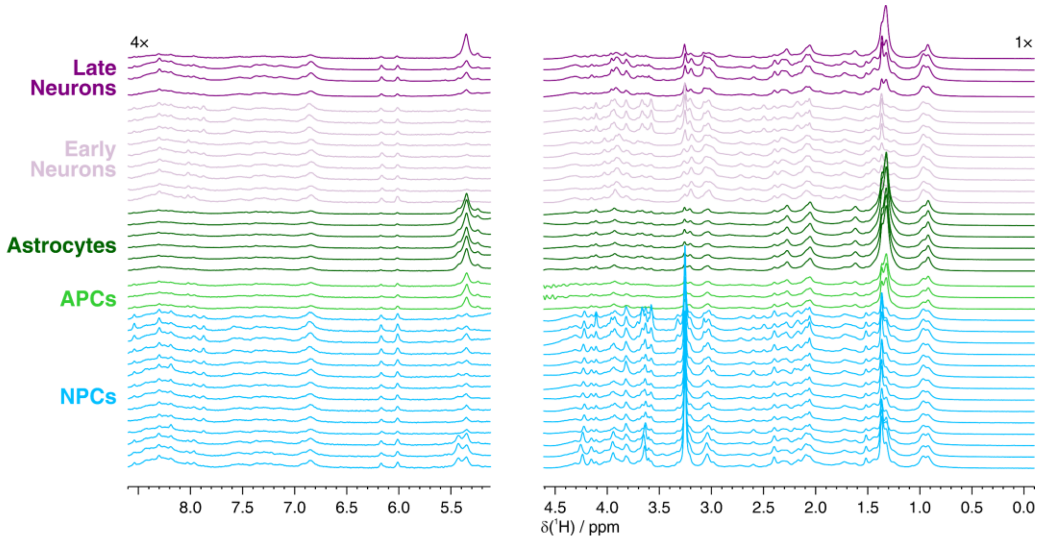


**Figure S8.** $^{1}H$ excitation sculpting NMR spectra collected at 950 MHz (22.3 T) on samples of neural progenitor cells (NPCs, light blue), astrocyte progenitor cells (APCs, light green), astrocytes (dark green), early neurons (pink), and late neurons (purple). The aromatic region (5.1-8.6 ppm) is scaled 4x for clarity.

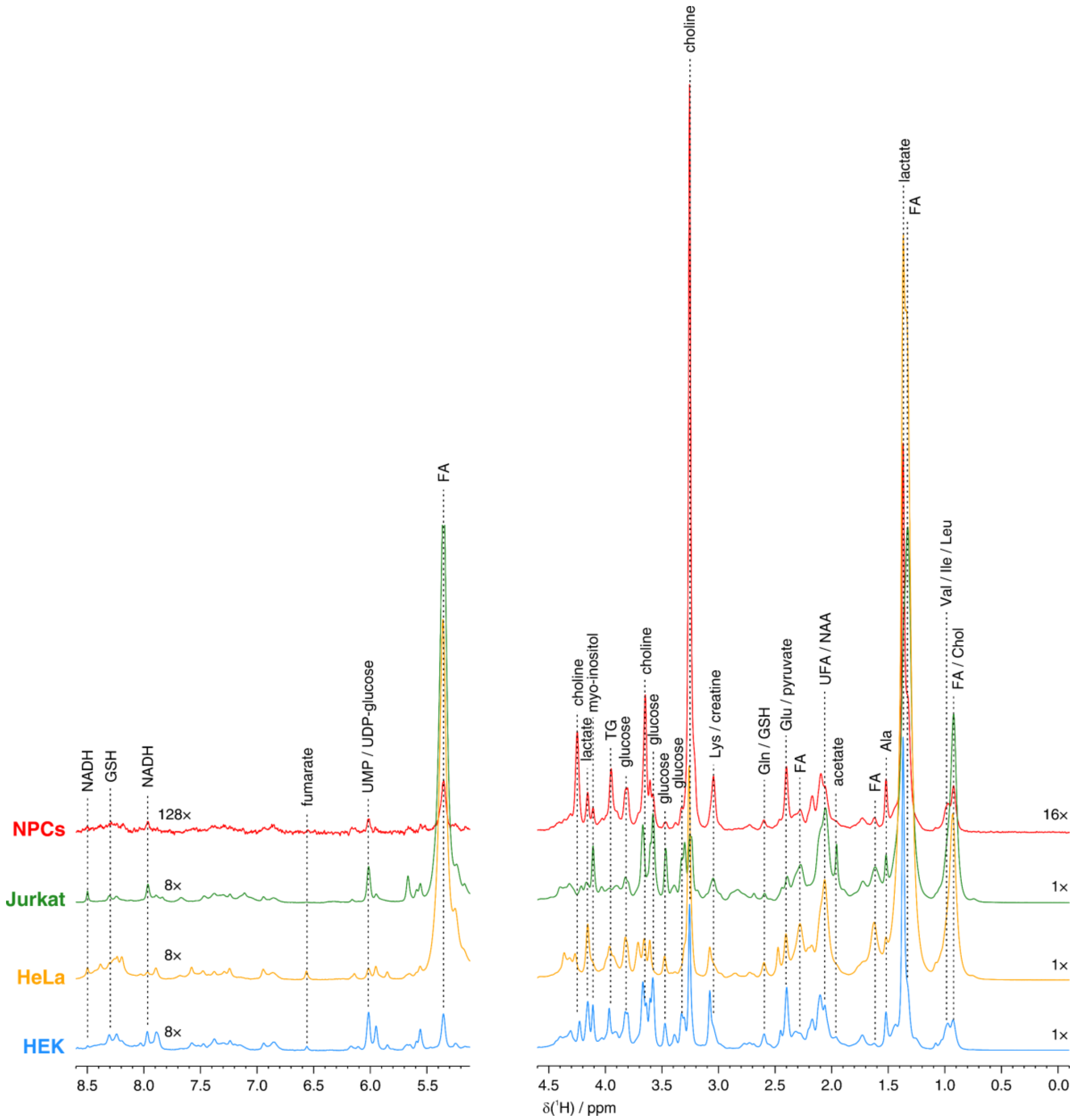


**Figure S9.** Tentative assignment of the most prominent spectral features overlaid to representative $^{1}$H CPMG NMR spectra of HEK293T (blue), HeLa (yellow), Jurkat T lymphocytes (green) and NPCs (red). Ala, alanine; Chol, cholesterol; FA, fatty acids; Gln, glutamine; Glu, glutamate; GSH, glutathione; Leu, leucine; Lys, lysine; Ile, isoleucine; NAA, N-acetyl aspartate; NADH, nicotinamide adenine dinucleotide; TG, triglycerides (glycerol); Val, valine; UFA, unsaturated fatty acids; UDP-glucose, uridine diphosphate glucose; UMP, uridine monophosphate.

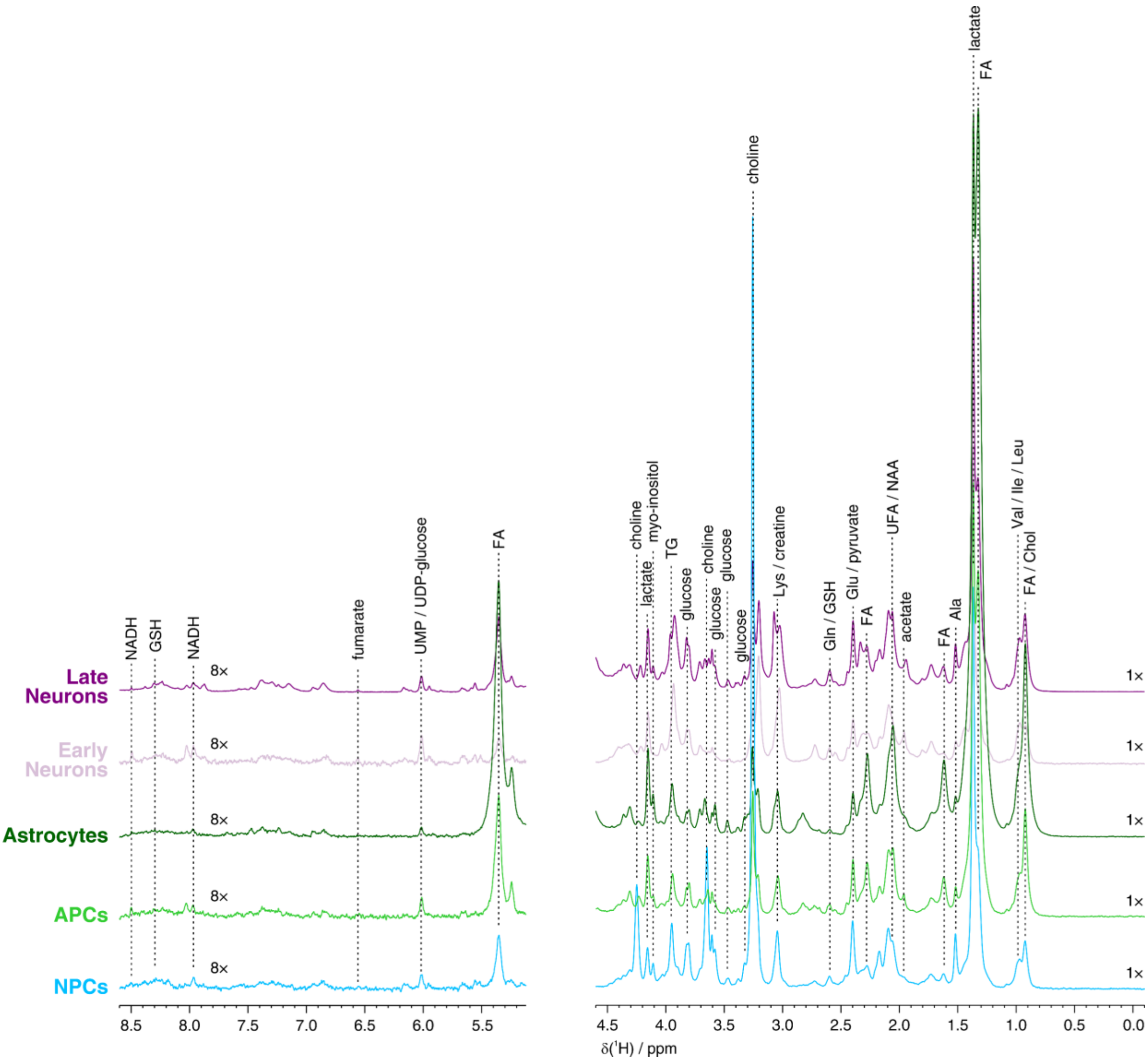


**Figure S10.** Tentative assignment of the most prominent spectral features overlaid to representative $^{1}$H CPMG NMR spectra of neural progenitor cells (NPCs, light blue), astrocyte progenitor cells (APCs, light green), astrocytes (dark green), early neurons (pink), and late neurons (purple). Ala, alanine; Chol, cholesterol; FA, fatty acids; Gln, glutamine; Glu, glutamate; GSH, glutathione; Leu, leucine; Lys, lysine; Ile, isoleucine; NAA, N-acetyl aspartate; NADH, nicotinamide adenine dinucleotide; TG, triglycerides (glycerol); Val, valine; UFA, unsaturated fatty acids; UDP-glucose, uridine diphosphate glucose; UMP, uridine monophosphate.

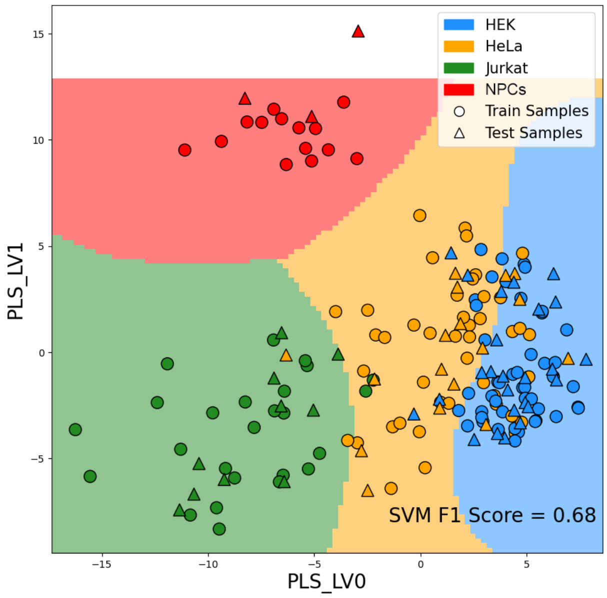


**Figure S11.** Projection of the first two components of the Partial Least Squares Discriminant Analysis (PLSDA), i.e., those which maximize the separation of different cell types, of the 4-class model trained on the $^{1}H$ excitation sculpting NMR spectra (see Figure S5 and S6), and classification margins as estimated by the Support Vector Machine (SVM). Size and shape of the margins are optimized through a stochastic grid search on SVM hyperparameters. In this scenario, the SVM model converged to a radial basis function kernel to optimally separate classes. Training (circles) and validation (triangles) samples and SVM boundaries are colored based on the class: HEK (blue), HeLa (yellow), Jurkat (green), and NPCs (red).

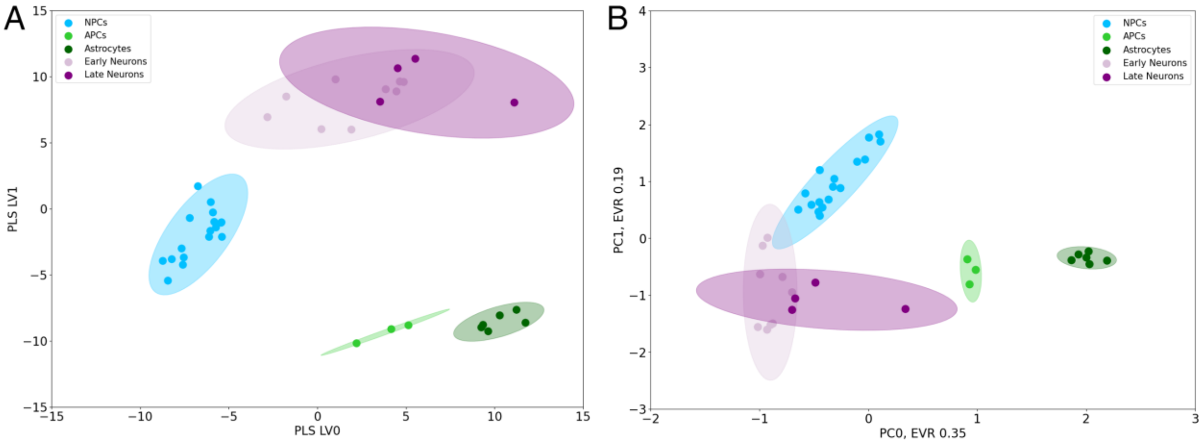


**Figure S12.** PLSDA (A) and PCA (B) analysis of $^{1}$H CPMG NMR spectra of a collection of CNS cell types. Samples are color-coded based on the stage of differentiation: neural progenitor cells (NPCs, light blue), early neurons (pink), late neurons (purple), astrocyte progenitor cells (APCs, light green), and mature astrocytes (dark green). 95% confidence ellipses calculated from the covariance matrix are shown. In part B, the explained variability ratio (EVR) for each PCA component is also shown.

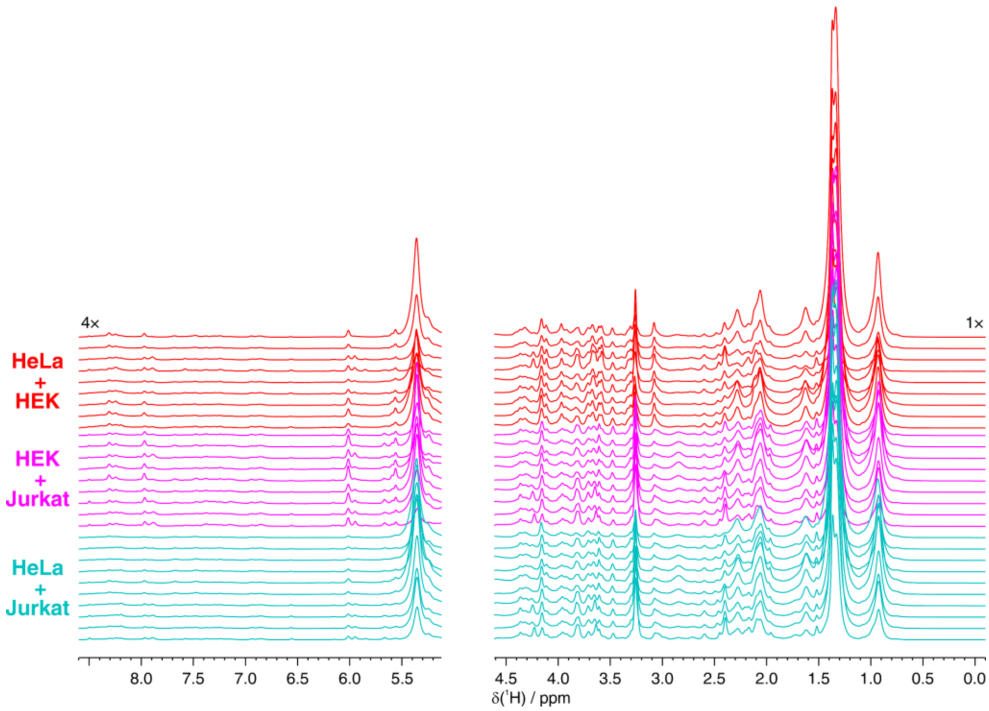


**Figure S13.** ¹H CPMG NMR spectra collected at 950 MHz (22.3 T) on mixed samples of HeLa + Jurkat (cyan), HEK + Jurkat (purple), and HeLa + HEK (red) cells. The aromatic region (5.1-8.6 ppm) is scaled 4x for clarity.

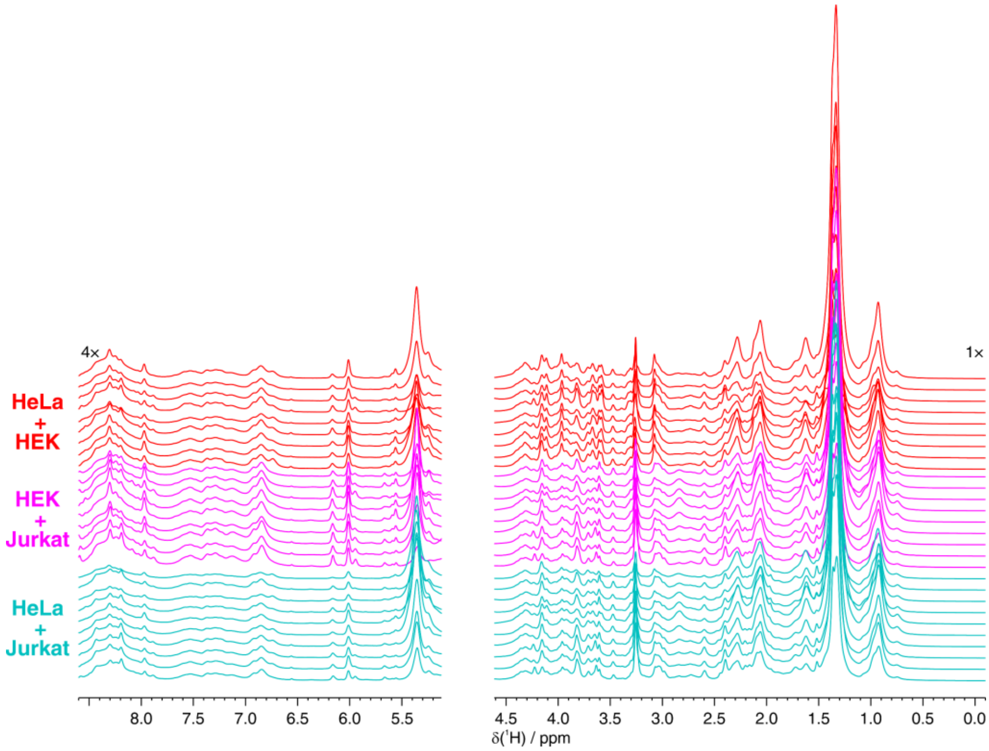


**Figure S14.** ¹H excitation sculpting NMR spectra collected at 950 MHz (22.3 T) on mixed samples of HeLa + Jurkat (cyan), HEK + Jurkat (purple), and HeLa + HEK (red) cells. The aromatic region (5.1-8.6 ppm) is scaled 4x for clarity.

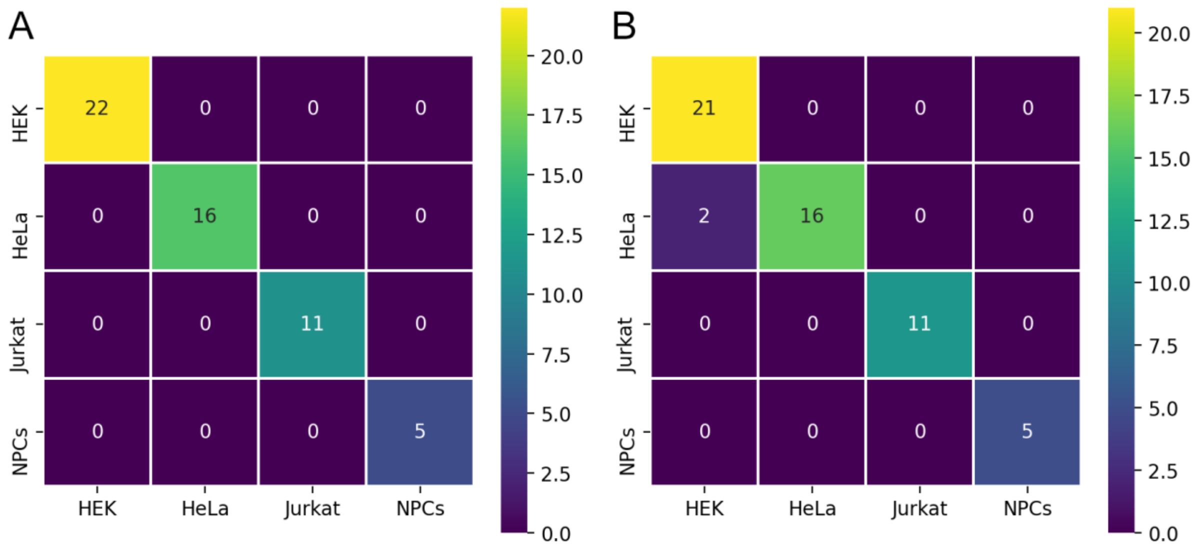


**Figure S15.** Confusion matrices of the Boruta + RFC model applied to the 4-class problem. (A) Model trained on the $^{1}$H CPMG NMR spectra. (B) Model trained on the $^{1}$H excitation sculpting NMR spectra. $F_1$ scores of 1.00 and 0.96 were obtained with the CPMG and the excitation sculpting spectra, respectively.

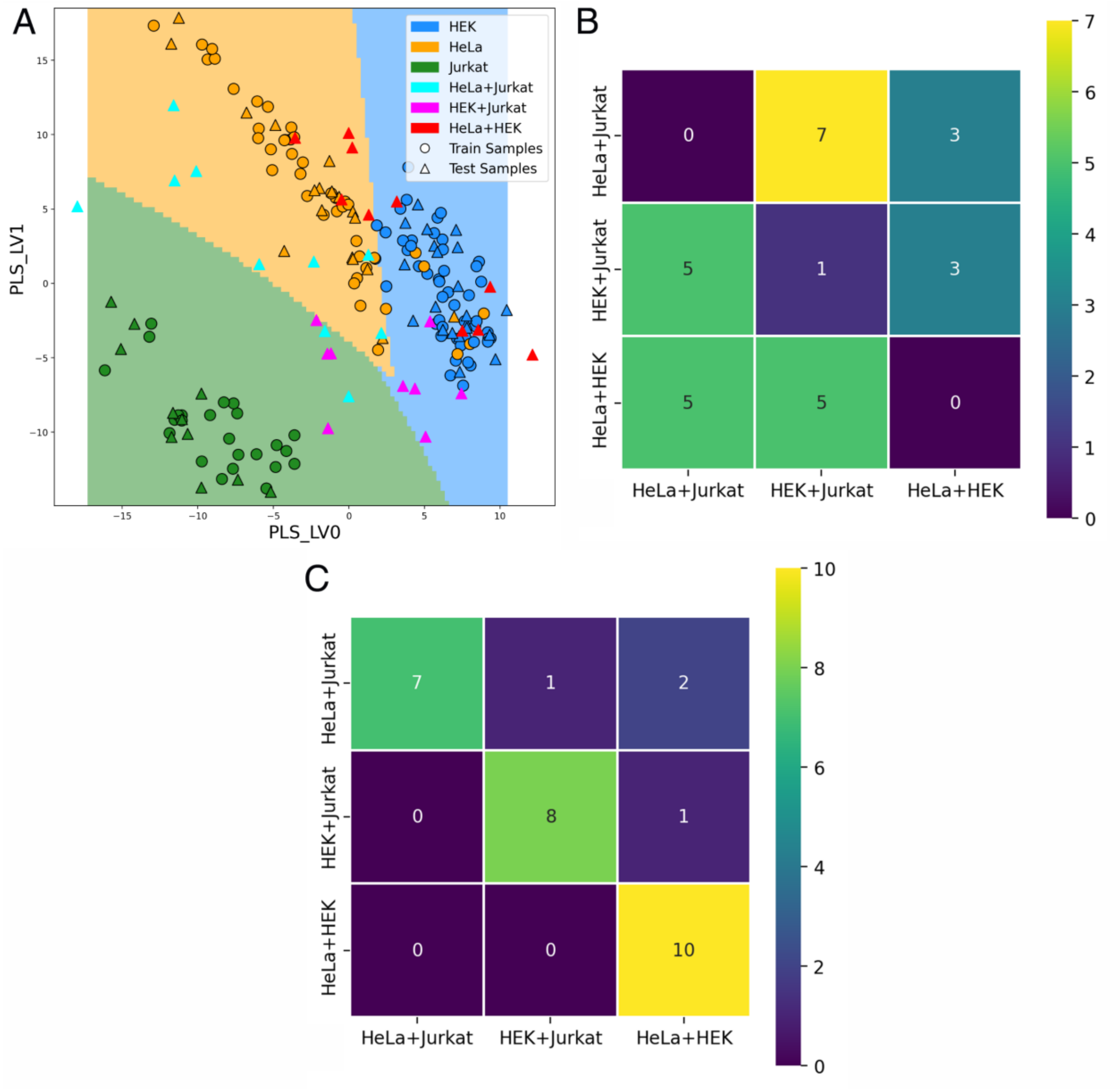


**Figure S16.** (A) Projection on the first two PLSDA components of the 3-class model trained on $^1$H excitation sculpting NMR spectra (see Figure S5 and S6) and classification margins as estimated by the SVM. Here, the SVM classifier automatically converges to a linear kernel function. Training (circles) and validation (triangles) samples and SVM boundaries are colored based on the class: HEK (blue), HeLa (yellow), and Jurkat (green). Mixed samples (triangles, see $^1$H excitation sculpting NMR spectra in Figure S14) are mapped on the PLS projection of the 3-class classifier and color-coded as follows: HeLa + Jurkat (cyan), HEK + Jurkat (purple), and HeLa + HEK (red). (B) Confusion matrix of the PLSDA + SVM model, trained on pure cell lines, used to predict mixed samples. This model results in a $F_1$ score of 0.08. (C) Confusion matrix of the Boruta + RFC model. This model results in a $F_1$ score of 0.85.

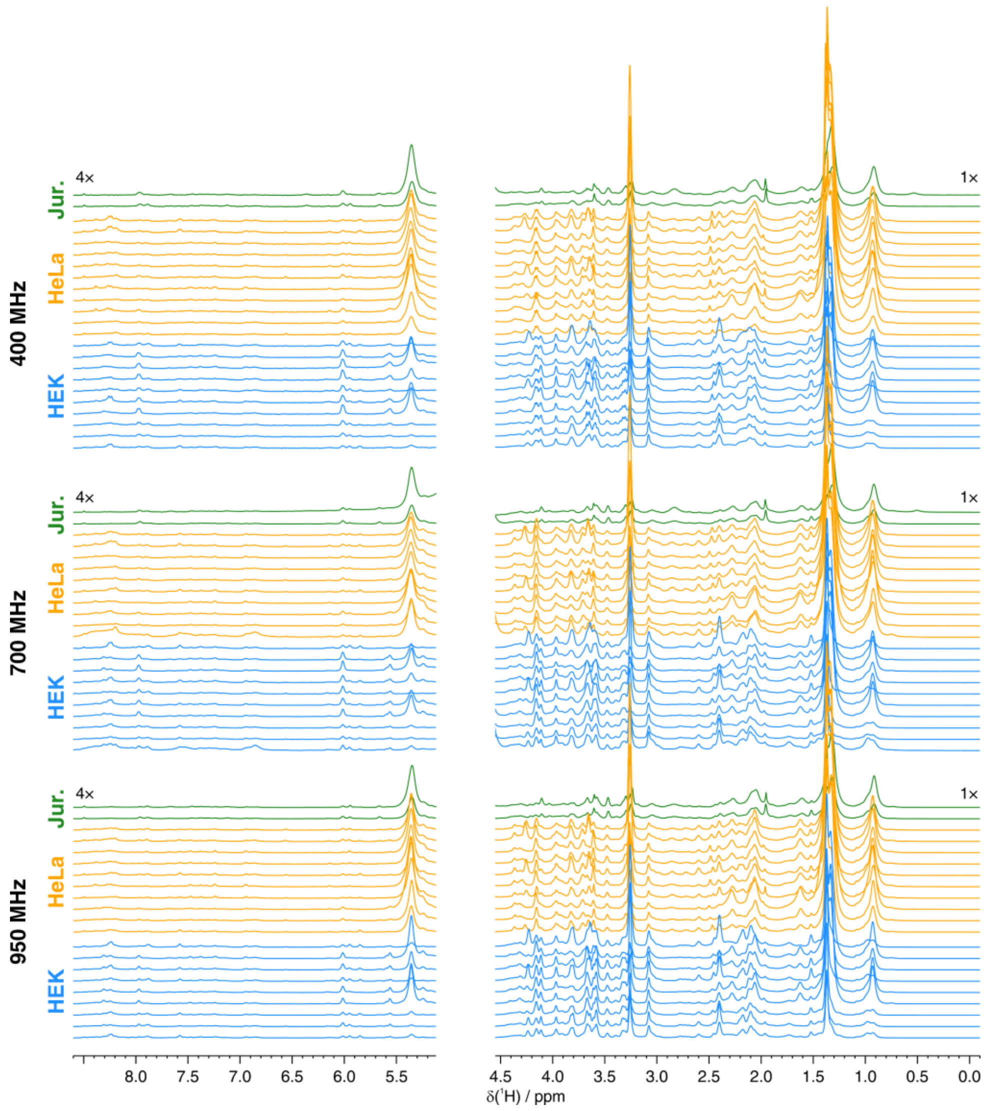


**Figure S17.** $^{1}$H CPMG NMR spectra collected at 950 MHz (22.3 T), 700 MHz (16.4 T), and 400 MHz (9.4 T) on samples of HEK293T (blue), HeLa (yellow), and Jurkat (green) cells. The aromatic region (5.1-8.6 ppm) is scaled 4x for clarity.

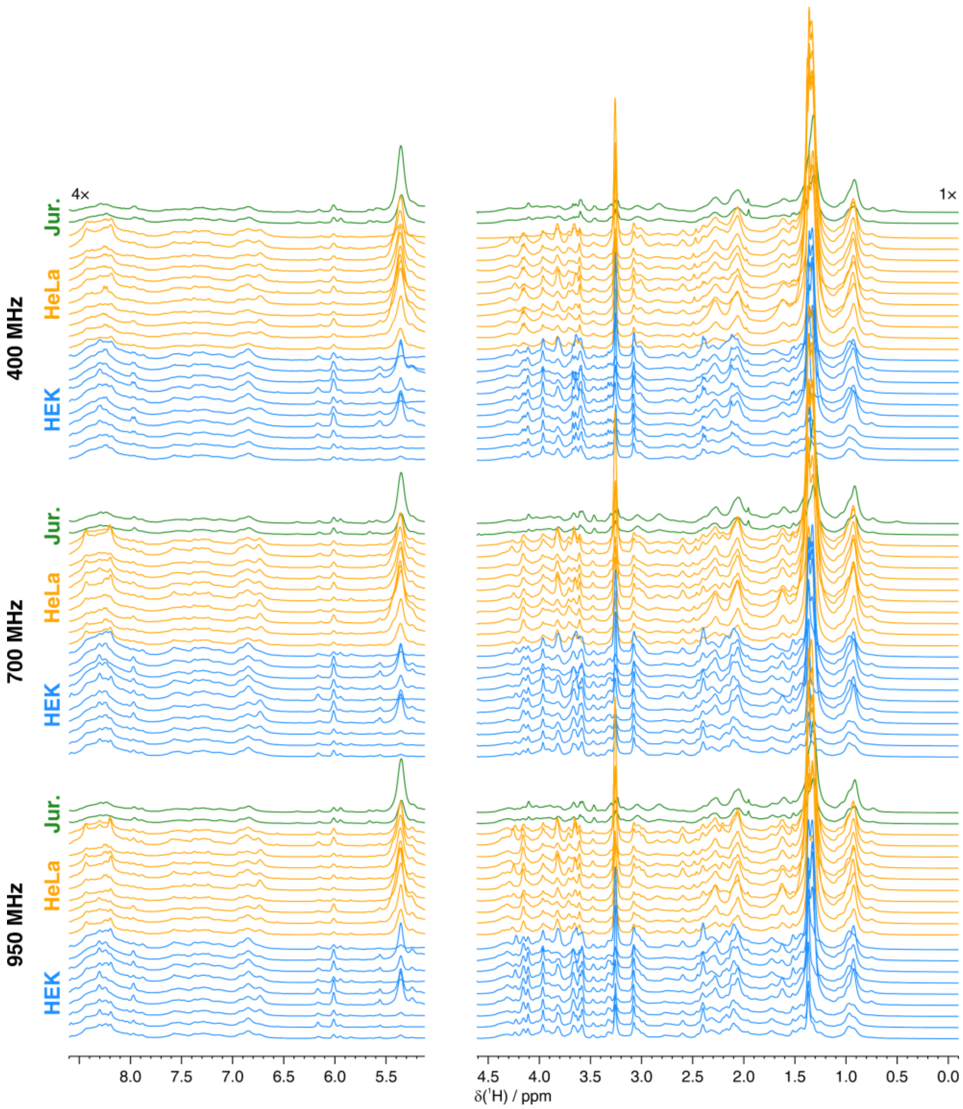


**Figure S18.** $^{1}$H excitation sculpting NMR spectra collected at 950 MHz (22.3 T), 700 MHz (16.4 T), and 400 MHz (9.4 T) on samples of HEK293T (blue), HeLa (yellow), and Jurkat (green) cells. The aromatic region (5.1-8.6 ppm) is scaled 4x for clarity.

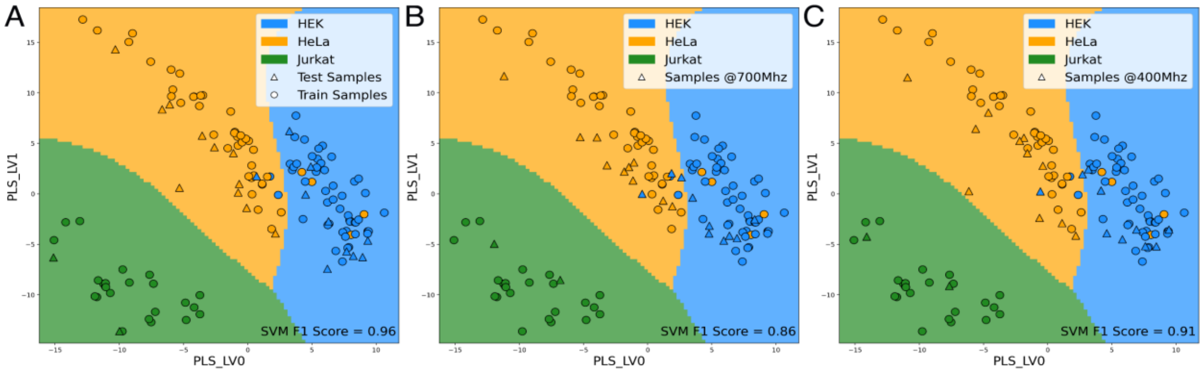


**Figure S19.** Classification of cell samples analyzed with $^{1}$H excitation sculpting NMR (see Figure S18) at (A) 950 MHz (22.3 T), (B) 700 MHz (16.4 T), and (C) 400 MHz (9.4 T) based on the 3-class PLSDA + SVD model trained with data acquired at 950 MHz. Training (circles) and validation (triangles) samples and SVM boundaries are colored based on the class: HEK (blue), HeLa (yellow), and Jurkat (green).

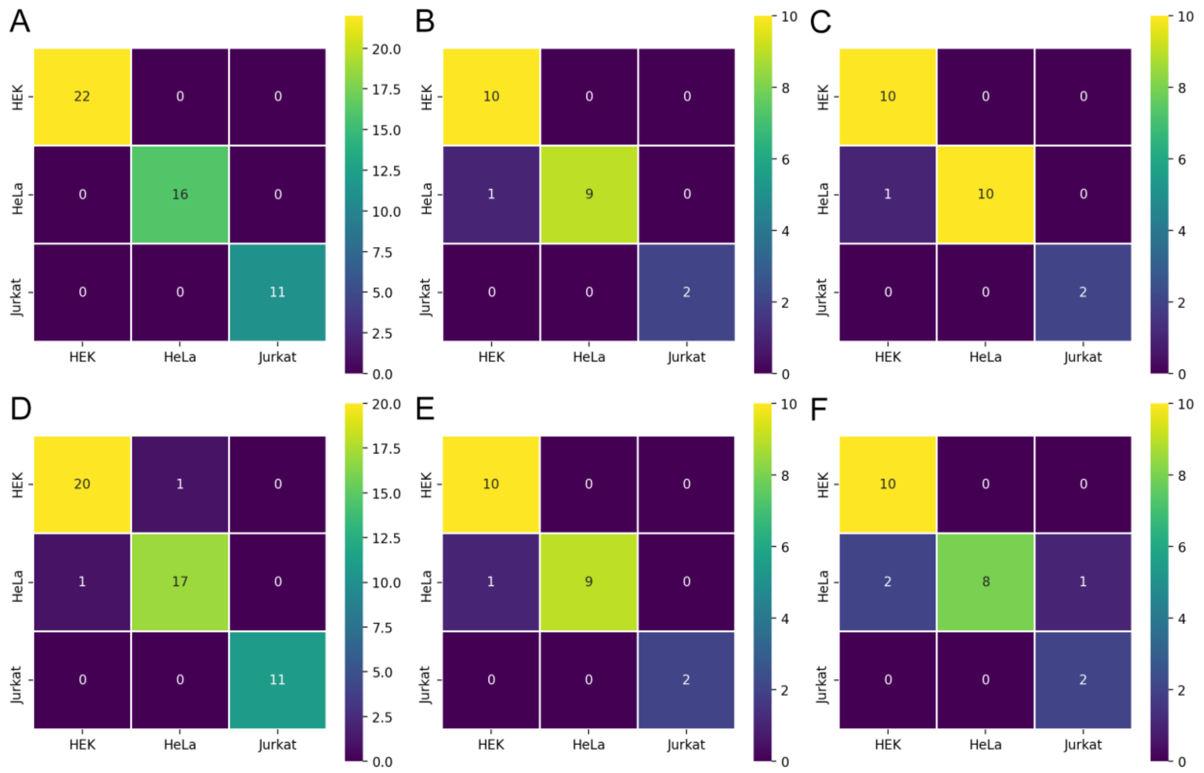


**Figure S20.** Classification of cell samples analyzed at decreasing magnetic fields by Boruta + RFC trained with data acquired at 950 MHz. Confusion matrices of the Boruta + RFC model applied on $^{1}H$ CPMG NMR spectra at (A) 950 MHz (22.3 T), (B) 700 MHz (16.4 T), and (C) 400 MHz (9.4 T), and on $^{1}H$ excitation sculpting NMR spectra at (D) 950 MHz (22.3 T), (E) 700 MHz (16.4 T), and (F) 400 MHz (9.4 T). The following $F_1$ scores were obtained: 1.00 (A), 0.95 (B), 0.96 (C), 0.96 (D), 0.95 (E), 0.87 (F).

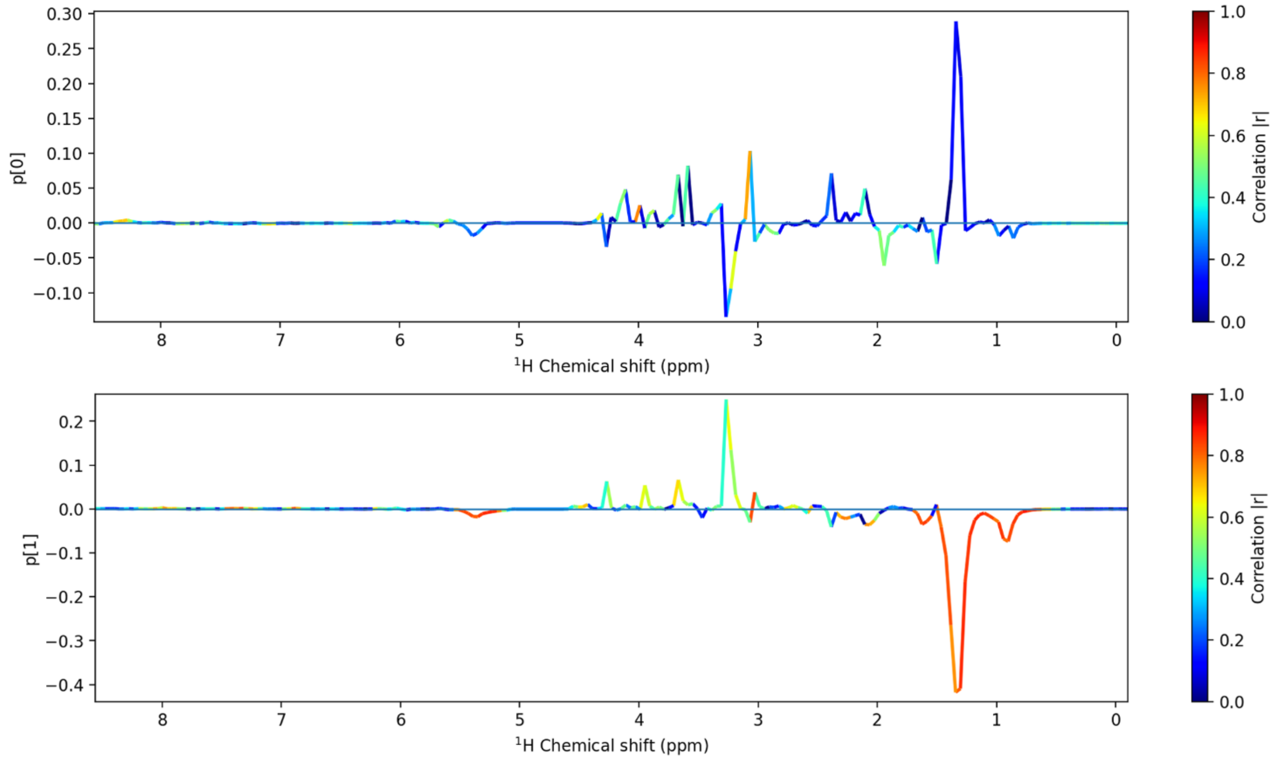


**Figure S21**. Coefficient-coded loading plot (SIMCA-P like) of first two latent variables (LV) of the 4-class PLSDA model (see Figure 2). Upper: LV0, lower: LV1. These plots give an overall perspective on how samples are projected in the 2D space, highlighting which spectral features are contributing the most in pushing a point left/right (LV0) and up/down (LV1). High loading intensity coupled with strong correlation with the scores in a specific direction is marking impactful spectral regions.

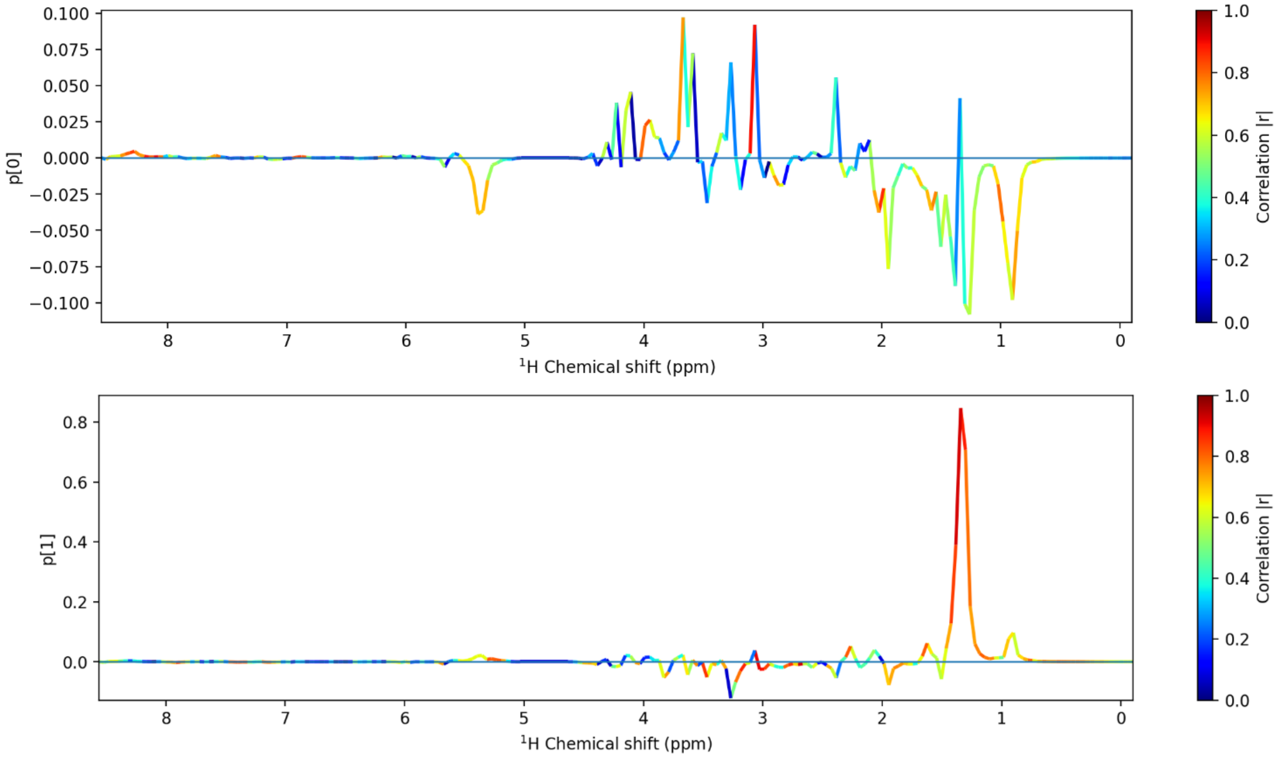


**Figure S22**. Coefficient-coded loading plot (SIMCA-P like) of first two latent variables (LV) of the 3-class PLSDA model (see Figure 4A). Upper: LV0, lower: LV1. These plots give an overall perspective on how samples are projected in the 2D space, highlighting which spectral features are contributing the most in pushing a point left/right (LV0) and up/down (LV1). High loading intensity coupled with strong correlation with the scores in a specific direction is marking impactful spectral regions.

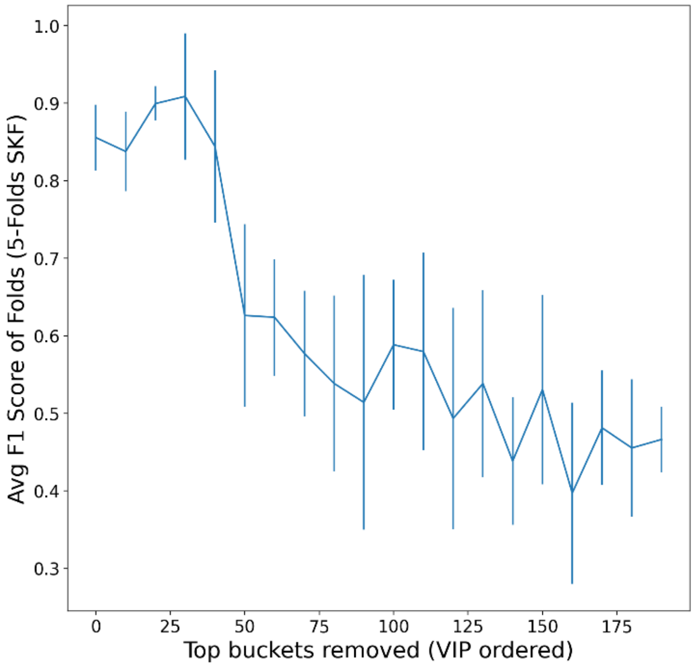


**Figure S23.** Analysis of sensitivity of PLSDA performance to the removal of VIP ranked buckets (4-class problem). Performance starts to drop significantly at the removal of the top 50 buckets, corresponding to 23% of the original spectral features.

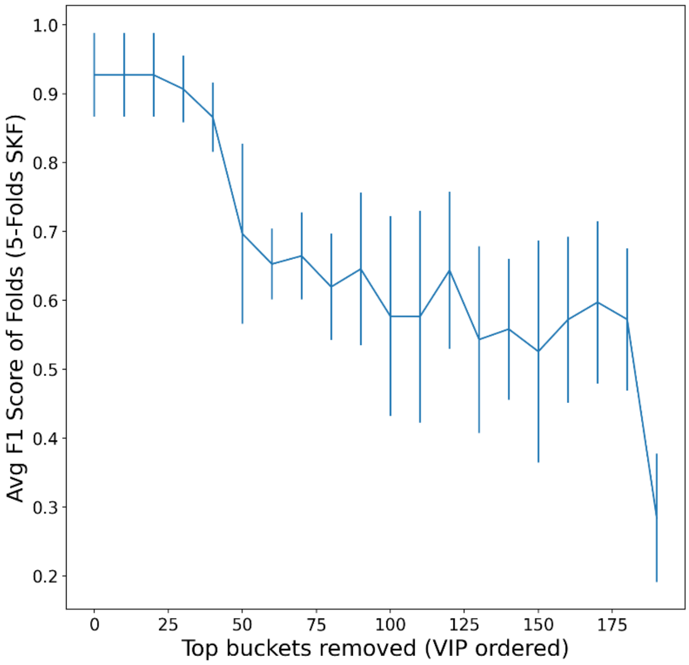


**Figure S24.** Analysis of sensitivity of PLSDA performance to the removal of VIP ranked buckets (3-class problem). Performance starts to drop significantly at the removal of the top 50 buckets, corresponding to 23% of the original spectral features.

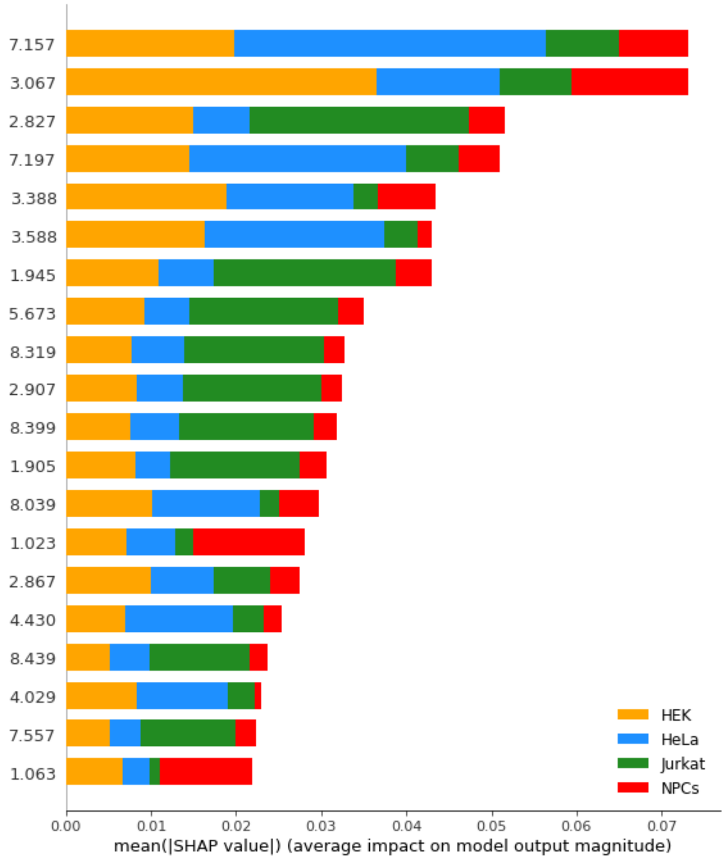


**Figure S25.** SHAP (SHapley Additive exPlanations) summary plot of the fitted Random Forest Classifier (RFC), 4-class problem. The top 20 Features are ranked according to their overall impact on the model output (average SHAP value), color-coded according to their class specificity.

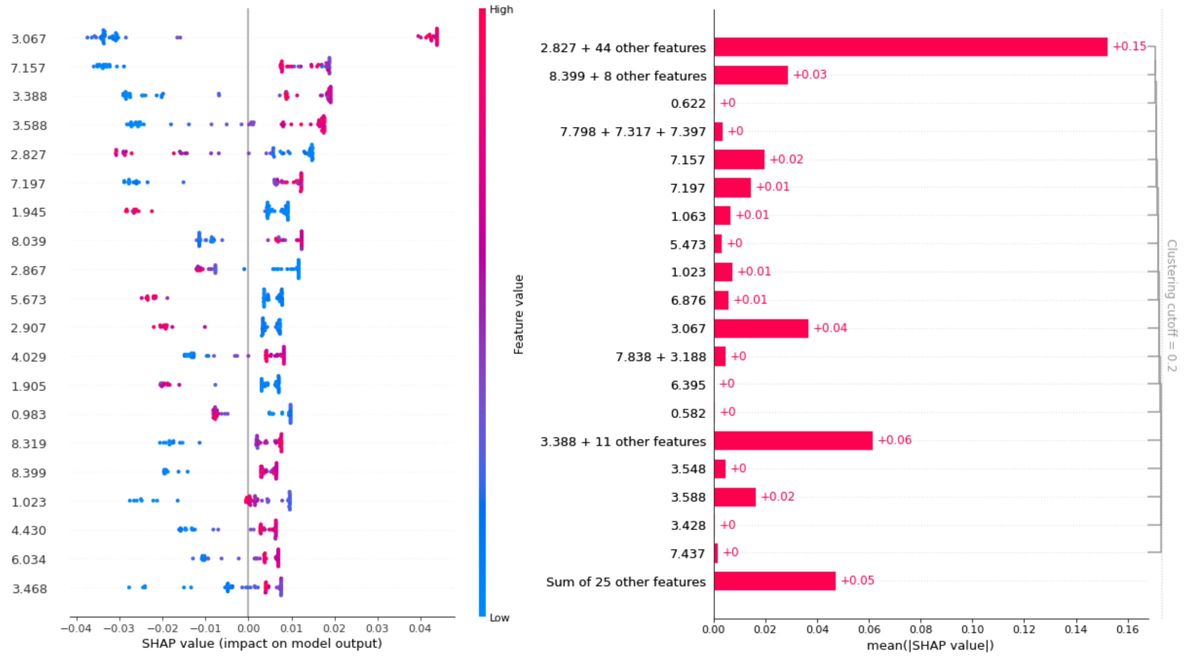


**Figure S26.** HEK class, 4-class problem SHAP Analysis. Beeswarm plot (left), barplot with feature interaction/redundancy (right). The beeswarm plot highlights relationships between feature values and SHAP values, i.e., their impact on model output. As an example, for class HEK, higher values in the spectral bucket at 3.067 ppm are pushing the decision toward the class. The barplot in the right panel summarizes how much of the impact in decision is due to single features or interactions amongst sets of features. For class HEK, spectral feature at 3.067 ppm is the single feature with the highest impact on class decision, but the majority of impact on decision is defined by the interaction/redundancy of feature 2.827 with 44 other spectral buckets. Interactions are computed using a model-agnostic Permutation Explainer, as implemented in https://github.com/shap.

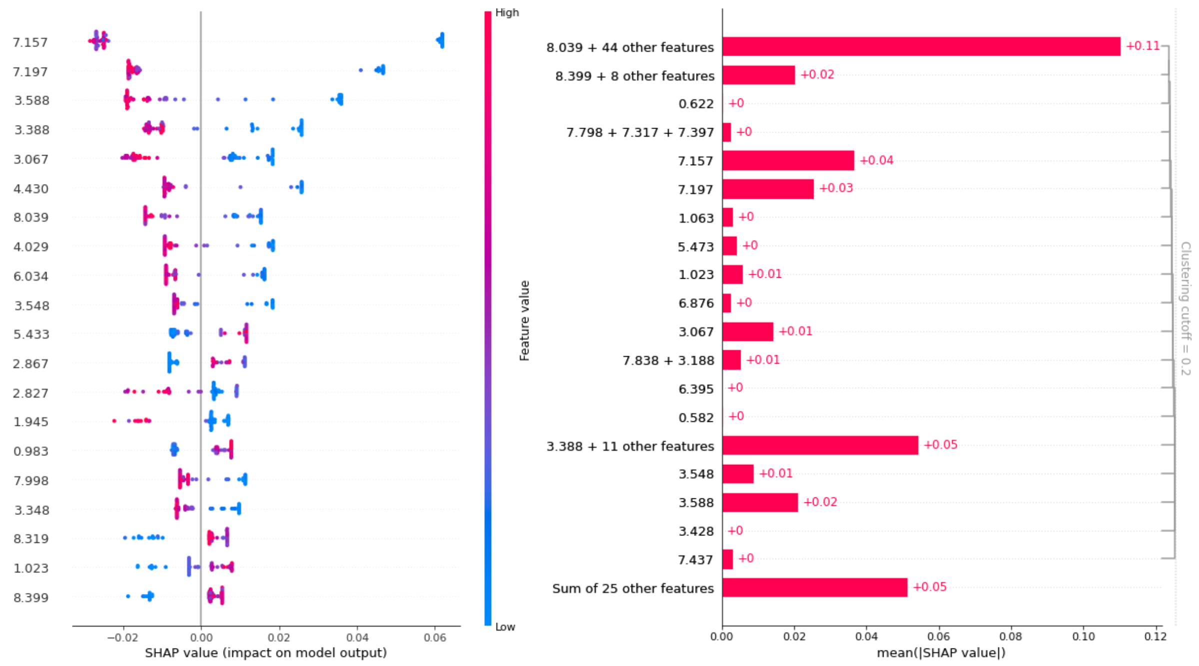


**Figure S27.** HeLa class, 4-class problem SHAP Analysis. Beeswarm plot (left), barplot with feature interaction/redundancy (right). The beeswarm plot highlights relationships between feature values and SHAP values, i.e., their impact on model output. As an example, for class HeLa, lower values in the spectral bucket at 7.157 ppm are pushing the decision toward the class. The barplot in the right panel summarizes how much of the impact in decision is due to single features or interactions amongst sets of features. For class HeLa, spectral feature at 7.157 ppm is the single feature with the highest impact on class decision, but the majority of impact on decision is defined by the interaction/redundancy of feature 8.039 with 44 other spectral buckets. Interactions are computed using a model-agnostic Permutation Explainer, as implemented in https://github.com/shap.

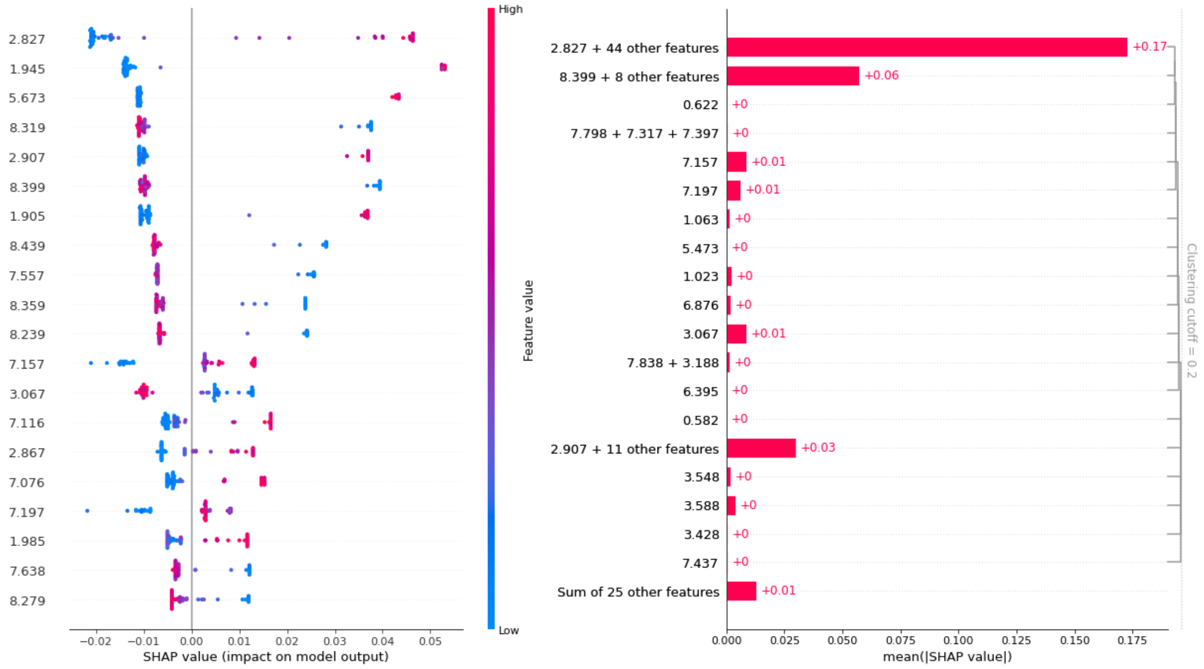


**Figure S28.** Jurkat class, 4-class problem SHAP Analysis. Beeswarm plot (left), barplot with feature interaction/redundancy (right). The beeswarm plot highlights relationships between feature values and SHAP values, i.e., their impact on model output. As an example, for class Jurkat, higher values in the spectral bucket at 2.827 ppm are pushing the decision toward the class. The barplot in the right panel summarizes how much of the impact in decision is due to single features or interactions amongst sets of features. For class Jurkat, spectral feature at 2.827 ppm is the single feature with the highest impact on class decision and its interaction/redundancy with 44 other spectral buckets account for the majority of impact on decision for the class. Interactions are computed using a model-agnostic Permutation Explainer, as implemented in https://github.com/shap.

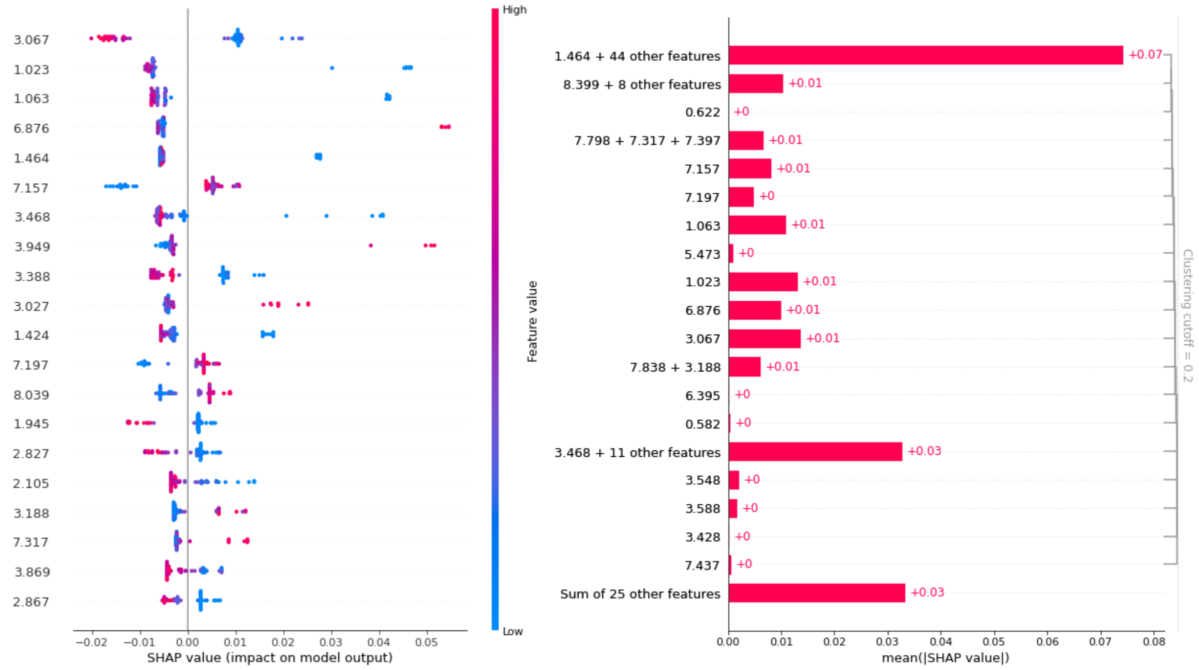


**Figure S29.** NPCs class, 4-class problem SHAP Analysis. Beeswarm plot (left), barplot with feature interaction/redundancy (right). The beeswarm plot highlights relationships between feature values and SHAP values, i.e., their impact on model output. As an example, for class NPCs, lower values in the spectral bucket at 1.023 and 1.063 ppm are pushing the decision toward the class. The barplot in the right panel summarizes how much of the impact in decision is due to single features or interactions amongst sets of features. For class NPCs, spectral features at 1.023 and 1.063 ppm are the single features with the highest impact on class decision, but most of impact on class decision comes from the interaction/redundancy of the bucket at 1.464 ppm with 44 other spectral buckets. Interactions are computed using a model-agnostic Permutation Explainer, as implemented in https://github.com/shap.

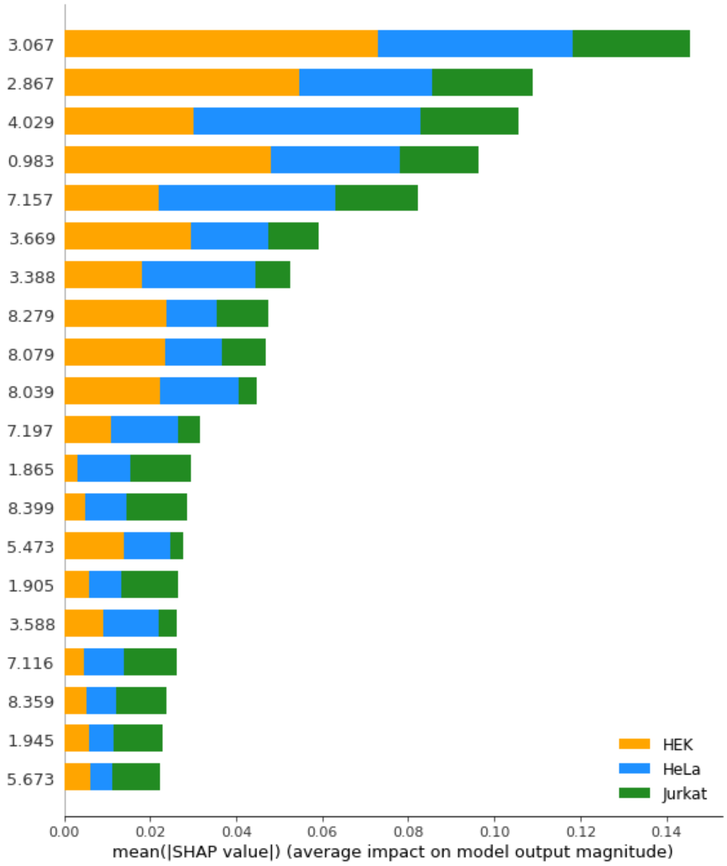


**Figure S30.** SHAP (SHapley Additive exPlanations) summary plot of the fitted Random Forest Classifier (RFC), 3-class problem. The top 20 Features are ranked according to their overall impact on the model output (average SHAP value), color-coded according to their class specificity.

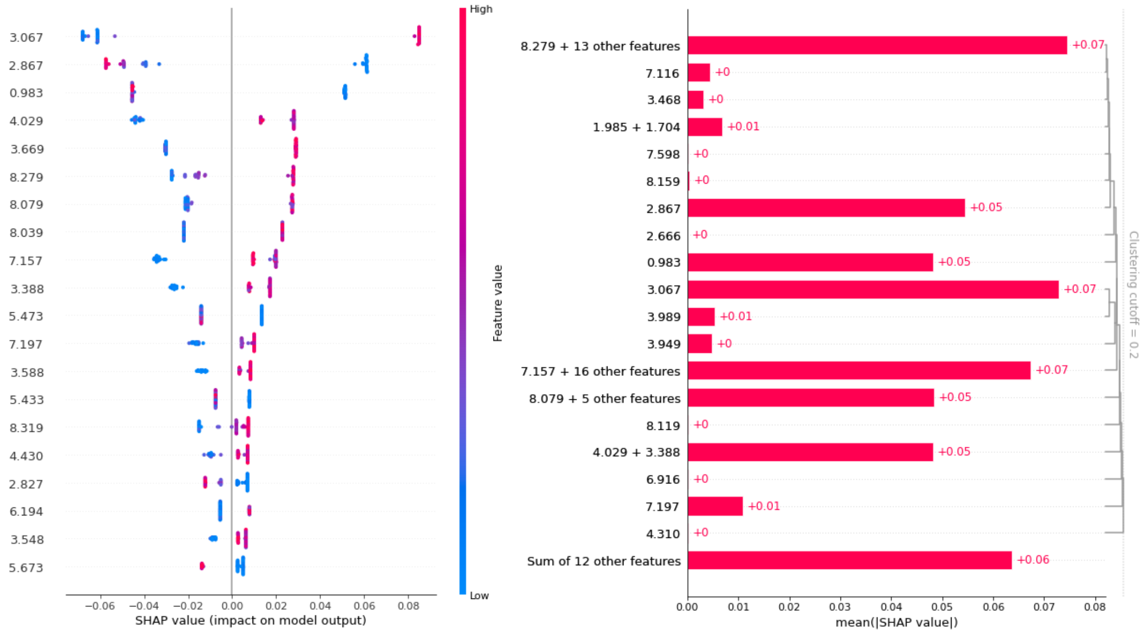


**Figure S31.** HEK class, 3-class problem SHAP Analysis. Beeswarm plot (left), barplot with feature interaction/redundancy (right). The beeswarm plot highlights relationships between feature values and SHAP values, i.e., their impact on model output.

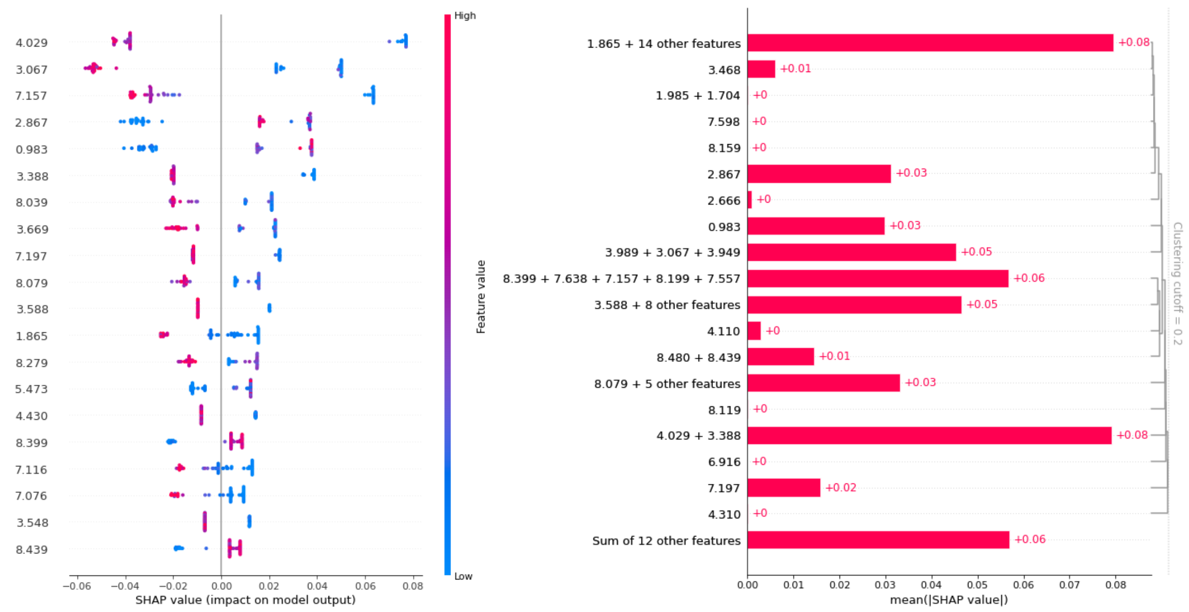


**Figure S32.** HeLa class, 3-class problem SHAP Analysis. Beeswarm plot (left), barplot with feature interaction/redundancy (right). The beeswarm plot highlights relationships between feature values and SHAP values, i.e., their impact on model output.

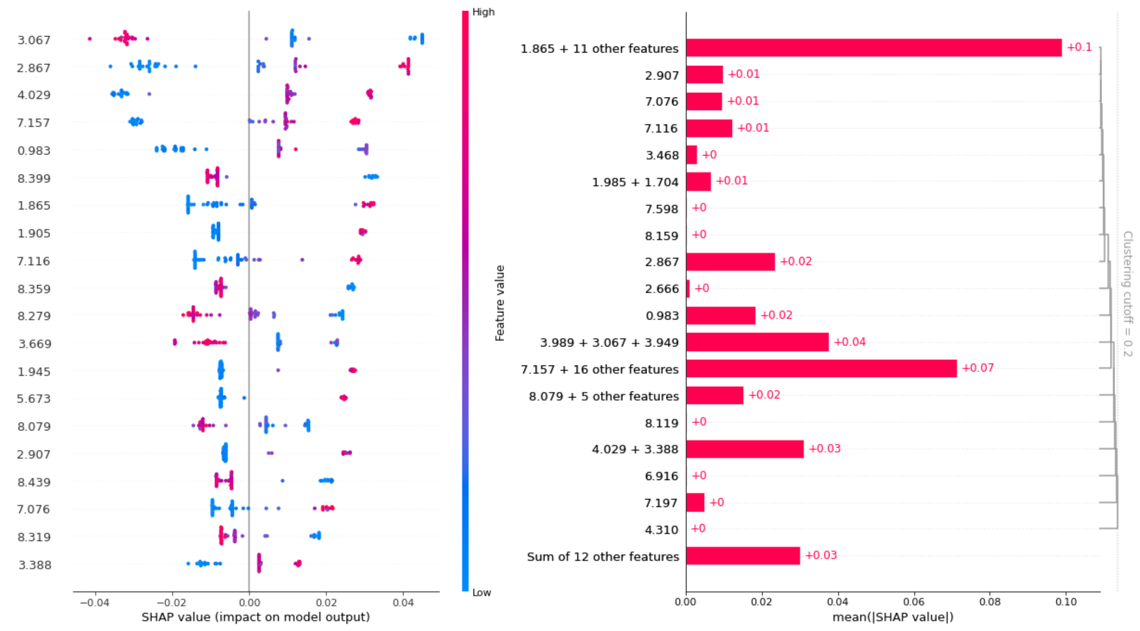


**Figure S33.** Jurkat class, 3-class problem SHAP Analysis. Beeswarm plot (left), barplot with feature interaction/redundancy (right). The beeswarm plot highlights relationships between feature values and SHAP values, i.e., their impact on model output.

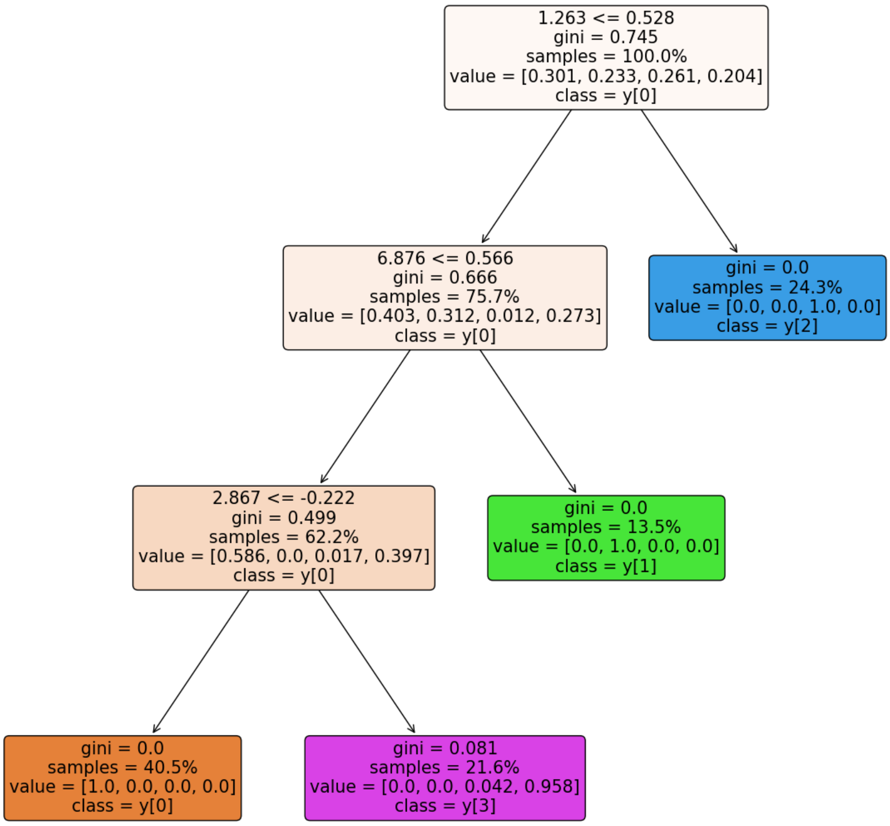


**Figure S34.** An example of the decision path of a tree inside the random forest ensemble. Each box represents a leaf in the tree. "n <= m": standardized threshold value at which the tree attempts to separate classes (n = feature name, m = threshold value); "gini": Gini impurity, i.e., a measure of the mixing of the classes in the leaf after the splitting; The lower the value, the higher number of a single class elements are separated in the leaf. "samples": percentage of the total dataset that is being split in the leaf. "value": fraction of samples of each class in the leaf after the split; "class": class decision performed in the leaf. The class with the highest fraction of samples wins the decision and the branching of the tree continues until the Gini impurity reaches 0. When Gini = 0, a single class is isolated in the leaf and the splitting stops for that branch of the tree.

## 2. Supplementary Tables

| | HEK, SR | HeLa, SR | Jurkat, SR | NPCs, SR | VIP |
|---|---|---|---|---|---|
| **8.359** | 0.894304 | 0.001128 | 2.523979 | 0.043011 | 1.507715 |
| **8.319** | 1.37481 | 0.029462 | 2.225682 | 0.064455 | 1.585503 |
| **8.279** | 1.433679 | 0.068705 | 1.319846 | 0.056423 | 1.484668 |
| **7.116** | 0.241894 | 0.442678 | 3.176411 | 0.032731 | 1.39998 |
| **7.076** | 0.468897 | 0.21255 | 2.634586 | 0.123402 | 1.44671 |
| **5.673** | 0.304709 | 0.276275 | 5.581721 | 0.014416 | 1.484406 |
| **4.11** | 1.131414 | 1.33146 | 0.078933 | 0.163511 | 1.350144 |
| **3.989** | 1.56914 | 0.027222 | 0.950141 | 0.150602 | 1.421223 |
| **3.869** | 1.406025 | 1.424711 | 0.058406 | 0.29661 | 1.38897 |
| **3.588** | 0.877457 | 2.677406 | 0.016643 | 0.162069 | 1.370228 |
| **3.348** | 1.079953 | 1.472045 | 0.053954 | 0.173225 | 1.336103 |
| **3.188** | 0.424676 | 0.216277 | 0.765468 | 0.50489 | 1.366711 |
| **3.067** | 4.476098 | 0.614096 | 0.469878 | 0.194982 | 1.553328 |
| **2.907** | 0.394767 | 0.101278 | 3.761487 | 0.009379 | 1.488165 |
| **2.867** | 0.648315 | 0.001589 | 1.839534 | 0.010406 | 1.471457 |
| **2.827** | 0.426572 | 0.014966 | 1.304915 | 0.009903 | 1.340093 |
| **1.945** | 0.272686 | 0.22858 | 7.709501 | 5.45E-05 | 1.489951 |
| **1.905** | 0.21582 | 0.325144 | 5.067297 | 0.001861 | 1.421384 |
| **1.865** | 0.217783 | 0.438366 | 3.137589 | 0.023471 | 1.384785 |
| **1.784** | 0.192296 | 0.267332 | 3.109759 | 0.002275 | 1.332246 |

**Table S1.** Top 20 PLSDA features in the 4-class problem, ranked by VIP (Variable Importance in Projection) score. The VIP score is a measure on how each feature contributes (via PLSDA weights) to the latent components by explaining variance in Y (the targets matrix). The higher, the more a feature globally impacts class discrimination. VIP > 1 is a common "above average importance" cutoff. Selectivity Ratios (SR) are also reported for each class. Selectivity ratios, defined as the ratio of predictive variance to orthogonal variance per feature, is a measure of how strongly a feature supports discrimination for a specific class.

| | HEK, SR | HeLa, SR | Jurkat, SR | VIP |
|---|---|---|---|---|
| **8.319** | 2.746261 | 0.273192 | 2.089247 | 1.623834 |
| **8.359** | 1.613246 | 0.156575 | 2.520296 | 1.555816 |
| **2.867** | 1.255786 | 0.105552 | 1.842092 | 1.506999 |
| **1.945** | 0.236583 | 0.053356 | 7.641173 | 1.469546 |
| **2.907** | 0.449654 | 4.96E-08 | 3.692061 | 1.468894 |
| **8.279** | 1.934778 | 0.164293 | 1.257885 | 1.461765 |
| **5.673** | 0.316156 | 0.00475 | 5.151212 | 1.453319 |
| **5.593** | 1.438583 | 0.945834 | 0.219364 | 1.450803 |
| **3.067** | 2.632047 | 0.397938 | 0.456503 | 1.428264 |
| **4.110** | 2.608239 | 2.840368 | 0.072154 | 1.423288 |
| **7.076** | 0.223454 | 0.038979 | 5.580428 | 1.401318 |
| **7.116** | 0.104191 | 0.150243 | 5.899932 | 1.391966 |
| **1.905** | 0.171213 | 0.065548 | 5.251193 | 1.391449 |
| **5.553** | 1.036159 | 0.703482 | 0.226961 | 1.388465 |
| **8.079** | 1.136228 | 0.062297 | 0.881257 | 1.370069 |
| **8.399** | 0.098487 | 0.149318 | 4.511611 | 1.363091 |
| **8.159** | 0.537859 | 0.000122 | 1.518776 | 1.362241 |
| **8.239** | 0.05416 | 0.19388 | 2.658084 | 1.361834 |
| **3.949** | 0.555304 | 0.000675 | 1.187518 | 1.35811 |
| **7.557** | 0.182266 | 0.06059 | 2.873928 | 1.354684 |

**Table S2.** Top 20 PLSDA features in the 3-class problem, ranked by VIP (Variable Importance in Projection) score. The VIP score is a measure on how each feature contributes (via PLSDA weights) to the latent components by explaining variance in Y (the targets matrix). The higher, the more a feature globally impacts class discrimination. VIP > 1 is a common "above average importance" cutoff. Selectivity Ratios (SR) are also reported for each class. Selectivity ratios, defined as the ratio of predictive variance to orthogonal variance per feature, is a measure of how strongly a feature supports discrimination for a specific class.

| feature | class | coef | se | ci_lower | ci_upper |
|---|---|---|---|---|---|
| **8.319** | HEK | 0.646488 | 0.039001 | 0.47868 | 0.814297 |
| **8.319** | HeLa | -0.04808 | 0.045959 | -0.24582 | 0.149665 |
| **8.319** | Jurkat | -0.49304 | 0.018162 | -0.57118 | -0.41489 |
| **8.319** | NPCs | -0.10537 | 0.011224 | -0.15366 | -0.05708 |
| **3.067** | HEK | 0.025219 | 0.002652 | 0.013806 | 0.036631 |
| **3.067** | HeLa | -0.00614 | 0.001369 | -0.01204 | -0.00025 |
| **3.067** | Jurkat | -0.0124 | 0.000936 | -0.01643 | -0.00837 |
| **3.067** | NPCs | -0.00667 | 0.000661 | -0.00951 | -0.00383 |
| **8.359** | HEK | 0.770201 | 0.043445 | 0.583271 | 0.95713 |
| **8.359** | HeLa | -0.01242 | 0.065001 | -0.2921 | 0.267252 |
| **8.359** | Jurkat | -0.64537 | 0.018425 | -0.72465 | -0.5661 |
| **8.359** | NPCs | -0.1124 | 0.005838 | -0.13752 | -0.08729 |
| **1.945** | HEK | -0.0149 | 0.001135 | -0.01979 | -0.01002 |
| **1.945** | HeLa | -0.00485 | 0.000603 | -0.00745 | -0.00226 |
| **1.945** | Jurkat | 0.01964 | 0.001116 | 0.014837 | 0.024442 |
| **1.945** | NPCs | 0.000114 | 0.000461 | -0.00187 | 0.002095 |
| **2.907** | HEK | -0.12103 | 0.007995 | -0.15543 | -0.08663 |
| **2.907** | HeLa | -0.02251 | 0.007732 | -0.05578 | 0.010756 |
| **2.907** | Jurkat | 0.133226 | 0.012808 | 0.078116 | 0.188335 |
| **2.907** | NPCs | 0.010321 | 0.00334 | -0.00405 | 0.024694 |
| **8.279** | HEK | 0.457514 | 0.033577 | 0.313042 | 0.601986 |
| **8.279** | HeLa | -0.05318 | 0.028777 | -0.177 | 0.070638 |
| **8.279** | Jurkat | -0.33082 | 0.028577 | -0.45378 | -0.20786 |
| **8.279** | NPCs | -0.07351 | 0.011873 | -0.12459 | -0.02242 |
| **5.673** | HEK | -0.19308 | 0.006186 | -0.21969 | -0.16646 |
| **5.673** | HeLa | -0.05983 | 0.011896 | -0.11102 | -0.00865 |
| **5.673** | Jurkat | 0.23071 | 0.005585 | 0.206678 | 0.254741 |
| **5.673** | NPCs | 0.022198 | 0.005706 | -0.00235 | 0.046747 |
| **2.867** | HEK | -0.09546 | 0.002497 | -0.10621 | -0.08472 |
| **2.867** | HeLa | 0.002004 | 0.004267 | -0.01635 | 0.020362 |
| **2.867** | Jurkat | 0.085944 | 0.005889 | 0.060606 | 0.111281 |
| **2.867** | NPCs | 0.007516 | 0.001826 | -0.00034 | 0.015371 |
| **7.076** | HEK | -1.61954 | 0.129953 | -2.17869 | -1.0604 |
| **7.076** | HeLa | -0.38062 | 0.078625 | -0.71891 | -0.04232 |
| **7.076** | Jurkat | 1.547264 | 0.020538 | 1.458897 | 1.635631 |
| **7.076** | NPCs | 0.452897 | 0.037367 | 0.29212 | 0.613675 |
| **1.905** | HEK | -0.04606 | 0.003351 | -0.06048 | -0.03164 |
| **1.905** | HeLa | -0.01846 | 0.002674 | -0.02997 | -0.00696 |
| **1.905** | Jurkat | 0.06228 | 0.00443 | 0.043221 | 0.081339 |
| **1.905** | NPCs | 0.002241 | 0.001456 | -0.00402 | 0.008505 |
| **3.989** | HEK | 0.088776 | 0.00167 | 0.08159 | 0.095961 |
| **3.989** | HeLa | -0.00636 | 0.006065 | -0.03246 | 0.019736 |
| **3.989** | Jurkat | -0.06018 | 0.00246 | -0.07076 | -0.04959 |
| **3.989** | NPCs | -0.02224 | 0.003506 | -0.03732 | -0.00716 |
| **7.116** | HEK | -0.90547 | 0.011912 | -0.95672 | -0.85422 |

| | | | | | |
|---|---|---|---|---|---|
| **7.116** | HeLa | -0.383 | 0.04775 | -0.58845 | -0.17755 |
| **7.116** | Jurkat | 1.114209 | 0.044457 | 0.922924 | 1.305494 |
| **7.116** | NPCs | 0.17426 | 0.066031 | -0.10985 | 0.458367 |
| **3.869** | HEK | 0.086596 | 0.003758 | 0.070429 | 0.102763 |
| **3.869** | HeLa | -0.03583 | 0.002538 | -0.04675 | -0.02491 |
| **3.869** | Jurkat | -0.01955 | 0.002402 | -0.02988 | -0.00922 |
| **3.869** | NPCs | -0.03122 | 0.002102 | -0.04026 | -0.02218 |
| **1.865** | HEK | -0.07095 | 0.003461 | -0.08584 | -0.05605 |
| **1.865** | HeLa | -0.03075 | 0.004149 | -0.0486 | -0.0129 |
| **1.865** | Jurkat | 0.089634 | 0.005079 | 0.067783 | 0.111486 |
| **1.865** | NPCs | 0.01206 | 0.001716 | 0.004677 | 0.019443 |
| **3.588** | HEK | 0.012528 | 0.000816 | 0.009015 | 0.016041 |
| **3.588** | HeLa | -0.00678 | 0.000473 | -0.00882 | -0.00474 |
| **3.588** | Jurkat | -0.00173 | 0.00053 | -0.00401 | 0.000545 |
| **3.588** | NPCs | -0.00401 | 0.000261 | -0.00514 | -0.00289 |
| **3.188** | HEK | -0.04567 | 0.001275 | -0.05116 | -0.04019 |
| **3.188** | HeLa | -0.00986 | 0.002385 | -0.02012 | 0.000401 |
| **3.188** | Jurkat | 0.033222 | 0.000393 | 0.03153 | 0.034913 |
| **3.188** | NPCs | 0.022312 | 0.000855 | 0.018631 | 0.025992 |
| **4.110** | HEK | 0.026691 | 0.000699 | 0.023683 | 0.029699 |
| **4.110** | HeLa | -0.01152 | 0.000663 | -0.01437 | -0.00867 |
| **4.110** | Jurkat | -0.00735 | 0.001301 | -0.01295 | -0.00175 |
| **4.110** | NPCs | -0.00782 | 0.001254 | -0.01322 | -0.00243 |
| **2.827** | HEK | -0.07446 | 0.00237 | -0.08466 | -0.06426 |
| **2.827** | HeLa | -0.00559 | 0.00426 | -0.02392 | 0.012738 |
| **2.827** | Jurkat | 0.073463 | 0.005674 | 0.049052 | 0.097874 |
| **2.827** | NPCs | 0.006587 | 0.001457 | 0.000317 | 0.012857 |
| **3.348** | HEK | 0.063487 | 0.006162 | 0.036973 | 0.090001 |
| **3.348** | HeLa | -0.02901 | 0.003276 | -0.0431 | -0.01492 |
| **3.348** | Jurkat | -0.01486 | 0.002682 | -0.0264 | -0.00332 |
| **3.348** | NPCs | -0.01962 | 0.002182 | -0.02901 | -0.01023 |
| **1.784** | HEK | -0.1229 | 0.013857 | -0.18252 | -0.06327 |
| **1.784** | HeLa | -0.04673 | 0.003956 | -0.06375 | -0.02971 |
| **1.784** | Jurkat | 0.162719 | 0.013918 | 0.102834 | 0.222605 |
| **1.784** | NPCs | 0.006909 | 0.010449 | -0.03805 | 0.051867 |

**Table S3.** Jackknife Confidence Intervals (CIs) for estimated regression coefficients (per class, per feature) of the top 20 VIP ranked features, in the 4-class problem. The Jackknife method implemented by the authors (similar to SIMCA-P implementation) allows to evaluate the stability of regression coefficients estimates by removing stratified subsets of samples. For the present case, coefficient SE (standard error) ad 95% CIs are estimated with a 5-fold stratified refitting (to ensure enough samples for each class are represented in each fold). Low SE, narrow CI and consistent sign (lower and upper CI bounds do not cross 0) define a coefficient that is stable under resampling.

| feature | class | coef | se | ci_lower | ci_upper |
|---|---|---|---|---|---|
| **8.319** | HEK | 0.684249 | 0.018904 | 0.602913 | 0.765584 |
| **8.319** | HeLa | -0.28004 | 0.029901 | -0.40869 | -0.15138 |
| **8.319** | Jurkat | -0.40421 | 0.011896 | -0.4554 | -0.35303 |
| **8.359** | HEK | 0.82838 | 0.05475 | 0.592808 | 1.063952 |
| **8.359** | HeLa | -0.28661 | 0.05285 | -0.51401 | -0.05922 |
| **8.359** | Jurkat | -0.54177 | 0.006686 | -0.57054 | -0.513 |
| **2.867** | HEK | -0.10351 | 0.000787 | -0.1069 | -0.10013 |
| **2.867** | HeLa | 0.031087 | 0.003854 | 0.014504 | 0.047669 |
| **2.867** | Jurkat | 0.072426 | 0.004494 | 0.053092 | 0.091761 |
| **1.945** | HEK | -0.01194 | 0.001211 | -0.01715 | -0.00673 |
| **1.945** | HeLa | -0.00428 | 0.000982 | -0.0085 | -5.6E-05 |
| **1.945** | Jurkat | 0.01622 | 0.000798 | 0.012788 | 0.019653 |
| **2.907** | HEK | -0.10867 | 0.006229 | -0.13547 | -0.08187 |
| **2.907** | HeLa | -3E-05 | 0.009356 | -0.04028 | 0.040224 |
| **2.907** | Jurkat | 0.108703 | 0.011509 | 0.059185 | 0.15822 |
| **8.279** | HEK | 0.418357 | 0.022968 | 0.319533 | 0.51718 |
| **8.279** | HeLa | -0.15019 | 0.001046 | -0.15469 | -0.14569 |
| **8.279** | Jurkat | -0.26817 | 0.022744 | -0.36602 | -0.17031 |
| **5.673** | HEK | -0.17013 | 0.005649 | -0.19444 | -0.14583 |
| **5.673** | HeLa | -0.01543 | 0.009078 | -0.0545 | 0.023625 |
| **5.673** | Jurkat | 0.18557 | 0.004999 | 0.164061 | 0.207079 |
| **5.593** | HEK | 0.543117 | 0.06646 | 0.257162 | 0.829071 |
| **5.593** | HeLa | -0.40034 | 0.054491 | -0.6348 | -0.16588 |
| **5.593** | Jurkat | -0.14278 | 0.025798 | -0.25377 | -0.03178 |
| **3.067** | HEK | 0.019048 | 0.002707 | 0.007403 | 0.030693 |
| **3.067** | HeLa | -0.00934 | 0.001828 | -0.0172 | -0.00147 |
| **3.067** | Jurkat | -0.00971 | 0.000957 | -0.01383 | -0.00559 |
| **4.110** | HEK | 0.031711 | 0.002366 | 0.021533 | 0.041889 |
| **4.110** | HeLa | -0.02575 | 0.001283 | -0.03127 | -0.02023 |
| **4.110** | Jurkat | -0.00596 | 0.0021 | -0.01499 | 0.003077 |
| **7.076** | HEK | -1.02487 | 0.075943 | -1.35163 | -0.69812 |
| **7.076** | HeLa | -0.31879 | 0.026824 | -0.43421 | -0.20337 |
| **7.076** | Jurkat | 1.343662 | 0.063867 | 1.068865 | 1.618459 |
| **7.116** | HEK | -0.53602 | 0.026722 | -0.651 | -0.42105 |
| **7.116** | HeLa | -0.42221 | 0.029779 | -0.55034 | -0.29408 |
| **7.116** | Jurkat | 0.958233 | 0.030061 | 0.828889 | 1.087576 |
| **1.905** | HEK | -0.0354 | 0.002634 | -0.04673 | -0.02407 |
| **1.905** | HeLa | -0.01534 | 0.002474 | -0.02598 | -0.00469 |
| **1.905** | Jurkat | 0.050738 | 0.003772 | 0.03451 | 0.066966 |
| **5.553** | HEK | 0.66139 | 0.064906 | 0.382124 | 0.940656 |
| **5.553** | HeLa | -0.4785 | 0.057694 | -0.72674 | -0.23026 |
| **5.553** | Jurkat | -0.18289 | 0.022335 | -0.27899 | -0.08679 |
| **8.079** | HEK | 1.847862 | 0.144201 | 1.227417 | 2.468307 |
| **8.079** | HeLa | -0.50078 | 0.130456 | -1.06209 | 0.060527 |
| **8.079** | Jurkat | -1.34708 | 0.044243 | -1.53744 | -1.15672 |

| | | | | | |
|---|---|---|---|---|---|
| **8.399** | HEK | 0.371003 | 0.021192 | 0.279819 | 0.462186 |
| **8.399** | HeLa | 0.330095 | 0.042303 | 0.148079 | 0.512111 |
| **8.399** | Jurkat | -0.7011 | 0.035804 | -0.85515 | -0.54704 |
| **8.159** | HEK | 1.737166 | 0.117906 | 1.229859 | 2.244473 |
| **8.159** | HeLa | -0.02688 | 0.08786 | -0.40492 | 0.351146 |
| **8.159** | Jurkat | -1.71028 | 0.036886 | -1.86899 | -1.55157 |
| **8.239** | HEK | 0.195644 | 0.070155 | -0.10621 | 0.497495 |
| **8.239** | HeLa | 0.294168 | 0.067948 | 0.001811 | 0.586525 |
| **8.239** | Jurkat | -0.48981 | 0.032386 | -0.62916 | -0.35047 |
| **3.949** | HEK | 0.045993 | 0.006994 | 0.015898 | 0.076087 |
| **3.949** | HeLa | -0.00163 | 0.005071 | -0.02345 | 0.020184 |
| **3.949** | Jurkat | -0.04436 | 0.00202 | -0.05305 | -0.03567 |
| **7.557** | HEK | 1.10841 | 0.152152 | 0.453752 | 1.763067 |
| **7.557** | HeLa | 0.536397 | 0.122143 | 0.01086 | 1.061934 |
| **7.557** | Jurkat | -1.64481 | 0.03255 | -1.78486 | -1.50476 |

**Table S4.** Jackknife Confidence Intervals (CIs) for estimated regression coefficients (per class, per feature) of the top 20 VIP ranked features, in the 3-class problem. The Jackknife method implemented by the authors (similar to SIMCA-P implementation) allows to evaluate the stability of regression coefficients estimates by removing stratified subsets of samples. For the present case, coefficient SE (standard error) ad 95% CIs are estimated with a 5-fold stratified refitting (to ensure enough samples for each class are represented in each fold). Low SE, narrow CI and consistent sign (lower and upper CI bounds do not cross 0) define a coefficient that is stable under resampling.
